# Endothelin Receptor Antagonists: Status Quo and Future Perspectives for Targeted Therapy

**DOI:** 10.3390/jcm9030824

**Published:** 2020-03-18

**Authors:** Frederik C. Enevoldsen, Jayashree Sahana, Markus Wehland, Daniela Grimm, Manfred Infanger, Marcus Krüger

**Affiliations:** 1 Department of Biomedicine, Aarhus University, Høegh-Guldbergsgade 10, 8000 Aarhus C, Denmark; f.enevold@hotmail.com (F.C.E.); jaysaha@biomed.au.dk (J.S.); dgg@biomed.au.dk (D.G.); 2 Clinic for Plastic, Aesthetic and Hand Surgery, Otto von Guericke University, Leipziger Str. 44, 39120 Magdeburg, Germany; markus.wehland@med.ovgu.de (M.W.); manfred.infanger@med.ovgu.de (M.I.)

**Keywords:** pulmonary arterial hypertension, cancer, renal disease, fibrotic disorders, systemic scleroderma, cerebral vasospasm, pain management, ambrisentan, atrasentan, bosentan, clazosentan, macitentan, zibotentan

## Abstract

The endothelin axis, recognized for its vasoconstrictive action, plays a central role in the pathology of pulmonary arterial hypertension (PAH). Treatment with approved endothelin receptor antagonists (ERAs), such as bosentan, ambrisentan, or macitentan, slow down PAH progression and relieves symptoms. Several findings have indicated that endothelin is further involved in the pathogenesis of certain other diseases, making ERAs potentially beneficial in the treatment of various conditions. In addition to PAH, this review summarizes the use and perspectives of ERAs in cancer, renal disease, fibrotic disorders, systemic scleroderma, vasospasm, and pain management. Bosentan has proven to be effective in systemic sclerosis PAH and in decreasing the development of vasospasm-related digital ulcers. The selective ERA clazosentan has been shown to be effective in preventing cerebral vasospasm and delaying ischemic neurological deficits and new infarcts. Furthermore, in the SONAR (Study Of Diabetic Nephropathy With Atrasentan) trial, the selective ERA atrasentan reduced the risk of renal events in patients with diabetes and chronic kidney disease. These data suggest atrasentan as a new therapy in the treatment of diabetic nephropathy and possibly other renal diseases. Preclinical studies regarding heart failure, cancer, and fibrotic diseases have demonstrated promising effects, but clinical trials have not yet produced measurable results. Nevertheless, the potential benefits of ERAs may not be fully realized.

## 1. Endothelin in Health and Disease

The endothelin axis is most recognized for its potent vasoconstrictive action involved in the physiological regulation of vascular tone. The overproduction of endothelin in the lung can cause pulmonary arterial hypertension (PAH). Thus, endothelin receptor antagonists (ERAs) have been approved for the treatment of this disease [1]. Beyond hypertensive pathologies, the endothelin axis has pleiotropic functions associated with fundamental cellular processes, including cell proliferation and apoptosis. Endothelin is autocrinally regulated by physiochemical factors such as blood flow, mechanical stretch, or pH [2,3,4], and triggers the production of growth factors [5,6,7,8]. The ubiquitous distribution of endothelin and its receptors implicates their involvement in a wide variety of physiological and pathological processes among different organ systems [9]. Chronic endothelin stimulation has been implicated in several human cardiovascular, inflammatory, fibrogenic and oncologic diseases [1,10]. Furthermore, endothelin plays putative roles in pathologies such as heart failure, renal insufficiency, septic shock, atherosclerosis, and hemorrhage-associated cerebrovascular conditions, and can worsen insulin resistance by impairing glucose uptake in skeletal muscles [11]. Consequently, pharmaceutical agents that act on the endothelin axis might be beneficial in the treatment of numerous and diverse diseases [1]. This review will discuss the use and benefits of ERAs in PAH and beyond.

## 2. Endothelin and the Canonical Endothelin Pathway

**Figure 1:**
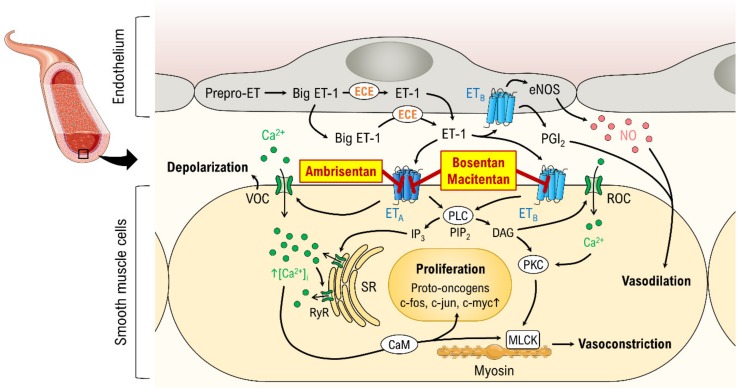
The canonical pathway of ET-1. Active ET-1 is synthesized from precursors via endothelin converting enzymes (ECE). ET-1 signals via two G protein-coupled receptors, ET_A_ and ET_B_, both of which share the same G_q/11_ signaling pathway. Activation of a receptor leads to the activation of phospholipase C (PLC), which processes the molecule phosphatidylinositol 4,5-bisphosphate (PIP_2_) into inositol trisphosphate (IP_3_) and diacylglycerol (DAG). IP_3_ binds its receptor located on the sarcoplasmic reticulum (SR), followed by a release of Ca^2+^ into the cytosol. Arrows describe cause–effect relationships. The right panel is a zoom-in (big arrow) of the box region. CaM, calmodulin; MLCK, myosin light-chain kinase; PKC, protein kinase C; ROC, receptor-operated channel; RyR, ryanodine receptor; VOC, voltage-gated channel. Parts of the figure are drawn using pictures from Servier Medical Art (https://smart.servier.com), licensed under a Creative Commons Attribution 3.0 Unported License (https://creativecommons.org/licenses/by/3.0).

The endothelin group comprises the three peptide isoforms, ET-1, ET-2, and ET-3, which have distinct tissue distributions. ET-1 is the most abundant isoform in the human cardiovascular system [9]. Under physiological conditions, active ET-1 is synthesized from precursors via endothelin converting enzymes predominantly in endothelial cells, but synthesis by vascular smooth muscle cells (VSMCs) has also been demonstrated [12]. ET-1 signaling occurs via the G protein-coupled receptors ET_A_ and ET_B_, both of which share the same G_q/11_ signaling pathway [13] (Figure 1). Activation of either receptor leads to the activation of phospholipase C, which processes the molecule phosphatidylinositol 4,5-bisphosphate into inositol trisphosphate and diacylglycerol. Inositol trisphosphate binds to its receptor, located on the sarco-/endoplasmic reticulum, followed by a release of Ca^2+^ into the cytosol [13] (Figure 1).

In the vascular system, ET_A_ receptors are mainly found in VSMCs. Their activation leads to vasoconstriction. In contrast, ET_B_ receptors are expressed in VSMCs and endothelial cells. Whereas ET_B_ receptor activation in smooth muscle mediates vasoconstriction, activation of the same receptor in endothelial cells causes vasodilation due to cytosolic Ca^2+^ binding to calmodulin to activate calmodulin kinase, which is responsible for phosphorylating endothelial nitric oxide synthase (NO) and thereby initiating NO synthesis [13,14]. Most vascular ET-1 degradation occurs intracellularly upon internalization of the ET-1 receptor complex [13].

## 3. Endothelin Receptor Antagonists (ERAs)

**Figure 2:**
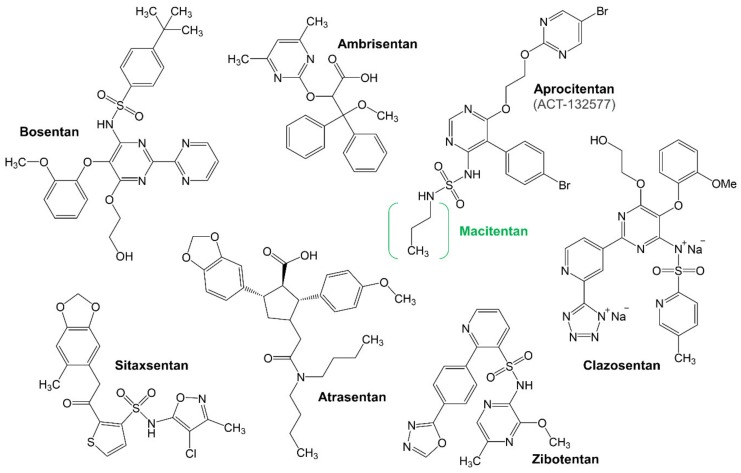
Chemical structures of ERAs.

Three different ERAs are used in the treatment of PAH: bosentan (Tracleer, RO470203), ambrisentan (Letairis, Volibris, LU-208075) and macitentan (Opsumit). In addition, several other potentially beneficial ERAs have been identified, including sitaxsentan (Thelin), clazosentan (RO-61-1790, VML-588, AXV-034), atrasentan (ABT-627, Xinlay), zibotentan (ZD4054), and aprocitentan (ACT-132577, the active metabolite of macitentan) (Figure 2).

### 3.1. Pharmacodynamics

Bosentan, ambrisentan, and macitentan are currently used in PAH treatment. Many synthetic molecules have been designed, tested, and evaluated for various conditions. These drugs can be divided into different categories because they are designed as either ET_A_-selective antagonists (with an ET_A_/ET_B_ selectivity ratio > 100) or dual ET_A_/ET_B_ antagonists (relatively equal selectivity between ET_A_ and ET_B_). Only a few ET_B_-selective antagonists have been developed, and these have never reached clinical use [1]. Other relevant ERAs that have been tested in human clinical studies on patients afflicted with PAH or other diseases are the ET_A_-specific compounds sitaxsentan [15], atrasentan [16], clazosentan [17], zibotentan [18,19], and aprocitentan (Table 1).

Bosentan is a non-peptide pyrimidine derivative that acts as a competitive, specific antagonist for both ET_A_ and ET_B_. It was the first ERA to be approved for the treatment of PAH in patients with a World Health Organization (WHO) functional class III–IV [33].

Ambrisentan is a propanoic acid derivative that is highly selective for ET_A_ [34]. Ambrisentan has theoretical benefits in terms of preventing cellular proliferation and vasoconstriction mediated by ET_A_ receptors on VSMCs while simultaneously preserving the vasodilator function of the ET_B_ receptor [35].

Macitentan belongs to the class of sulfamides and, like bosentan, it is a dual ERA, and thus inhibits ET-1 binding to both ET_A_ and ET_B_ [28]. At the cellular level, the drug differs from bosentan and ambrisentan through its binding profile, namely its slow receptor dissociation characteristics [36]. Due to this quality, macitentan theoretically has the potential to block ET-1-induced signaling more effectively than other ERAs [37].

### 3.2. Pharmacokinetics

Bosentan is an oral drug with a usual dosage of 125 mg twice daily after a titration period. The bioavailability is approximately 50%, and following oral administration, bosentan reaches its peak plasma concentration (C_max_) in healthy subjects after 3–5 h, with approximately 98% bound to albumin [35]. The former parameters are not significantly altered depending on food intake [33]. Steady state concentrations are reached in 3–5 days after administration of multiple doses. The volume of distribution is 30 L [20].

Bosentan mainly undergoes hepatic metabolism followed by almost complete elimination in the bile. The metabolism involves cytochrome P450 (CYP) 3A4 and CYP2C9. Three metabolites have been identified; one is active and contributes to the total response following the administration [35]. The drug has a terminal half-life of 5.4 h after oral administration [20].

Ambrisentan is administrated orally, with a usual dosage of 5 or 10 mg once daily. The absolute bioavailability is unknown. The drug is rapidly absorbed and reaches its C_max_ after approximately 1.5–2 h. Ninety-nine per cent is bound to plasma proteins. These parameters are not significantly affected or extended by food intake [38]. Steady state concentrations are achieved after four days of repeated administration [39]. The volume of distribution is unknown [40]. Ambrisentan undergoes hepatic metabolism. The drug is metabolized by glucuronidation and oxidative metabolism (primarily by CYP3A4). The main metabolites are ambrisentan glucuronide and 4-hydroxymethyl ambrisentan [39]. The drug has a terminal half-life of 9–15 h depending on the dosage [39]. The elimination of an oral dosage is predominantly by non-renal pathways, with 66% being recovered in the feces and only 22.6% in the urine [38].

Macitentan is administrated orally once daily, with a usual dosage of 10 mg [41]. The drug is slowly absorbed, reaching C_max_ approximately 8 h post-dose, and more than 99% is bound to plasma proteins [37]. Although the bioavailability is unknown, data from physiologically based pharmacokinetic modelling indicates high oral bioavailability. These parameters are not altered by food intake [28]. A steady state concentration is achieved in three days. The volume of distribution is estimated to be 40–50 L [42]. Macitentan undergoes hepatic metabolism by CYP450 enzymes, primarily CYP3A4, and to a lesser extent CYP2C9, CYP2C8 and CYP2C19 [28]. Macitentan has one active metabolite, ACT-132577 (also referred as aprocitentan), which is also an ET_A_/ET_B_ receptor antagonist [36]. Macitentan has a terminal half-life of 16 h [37], before the drug and its metabolites are excreted in the feces (24%) and urine (50%) [28,37].

### 3.3. Interactions and Contraindications

Due to the metabolism of bosentan, other drugs that are metabolized may induce or inhibit the specific CYP enzymes and can interact with this ERA. Some examples are ketoconazole, ciclosporin, simvastatin, and warfarin among many others [20,43]. Bosentan induces CYP2C9, CYP2C19 and CYP3A4, and because many contraceptives are metabolized by CYP3A4, there is a possibility of failure when co-administrating bosentan [20]. Dose-dependent increases in liver amino-transferase levels mean that, when possible, bosentan should be avoided in patients with moderate to severe hepatic impairment [43]. Bosentan is likely teratogenic, and pregnancy must be excluded before the start of treatment and prevented thereafter [20].

Drugs that affect the CYP enzymes may induce potential drug-drug interactions, and caution is advised when co-administrating ambrisentan with ciclosporin, ketoconazole or omeprazole. The same advice is applicable for inhibitors or inducers of P-glycoprotein (P-gp), UDP-glucuronosyltransferase and organic-anion-transporting polypeptide (OATP) [39]. Ambrisentan is not recommended for patients with severe hepatic impairment due to the hepatic and biliary involvement in its metabolism and excretion [38].

Given that macitentan is metabolized by several CYP enzymes, drugs that induce or inhibit these enzymes can affect its pharmacokinetics [36]. Macitentan is not a substrate of P-gp, and hepatic uptake is not dependent on OATP transport. Consequently, there is no accumulation in the liver and fewer possible drug-drug interactions [36]. Macitentan is contraindicated in pregnant women due to the risk of causing birth defects [37]. Patients with severe hepatic dysfunction or elevated liver enzymes should not be treated with macitentan [41].

### 3.4. Adverse Effects

The number of adverse effects (AE) from ERAs are dose-dependent. The general side effects are related to the vasodilator properties, including flushing, nausea, headache, nasal congestion and peripheral edema, as well as hypotension and palpitations [35]. Bosentan is associated with a reversible, dose-dependent elevation in aminotransferases [44]. Reduced hemoglobin levels and anemia can appear during ERA treatment [35]. Peripheral edema can also be observed with the use of ERAs [45,46]. Due to teratogenic effects in animal studies, all three ERAs are considered teratogenic and therefore contraindicated in pregnant women [35,47]. The type of contraception used during ERA treatment is essential, especially with regard to bosentan. Estroprogestative contraception is unreliable due to a powerful induction of CYP2C9 and CYP3A4 [20,35]. Cases of severe hepatitis-like drug reactions and even fatal liver injury have been related to the use of sitaxsentan, which led to its withdrawal in 2010 [48].

## 4. Pulmonary Arterial Hypertension (PAH)

PAH is a disease that affects the small arteries in the lungs. It is characterized by a vascular obstruction that leads to a progressive increase in vascular resistance and several vascular changes [49]. PAH is asymptomatic in early stages of the disease, but the symptoms, including persistent dyspnoea related to a decline in right heart function, appear in relation to disease progression [49,50]. If left untreated, the hemodynamic abnormalities ultimately result in a limitation of cardiac output and right ventricular failure [51]. Currently, PAH is not curable. However, pharmacological treatment can slow down the disease progression and relieve symptoms.

The pathogenesis of PAH is complex and likely involves multiple pathways rather than a few mechanisms. These pathways include the endothelin pathway, and patients with PAH present with elevated endothelin production [52].

Over the past few decades, several medications have been developed, and the current treatment is focused on three molecular signaling pathways: the prostacyclin, NO, and endothelin pathways [53]. The three approved ERAs, namely bosentan, ambrisentan, and macitentan, target the endothelin pathway and stimulate vasodilation, albeit with distinctive characteristics. The ERAs have been evaluated in several studies. The following trials are the most comprehensive studies. In the BREATHE-1 trial (Bosentan Randomized Trial of Endothelin Antagonist Therapy), bosentan significantly improved the 6-minute walking distance (6MWD) in PAH patients, with a mean difference (MD) of 44 m compared to the placebo group [54]. Similarly, in the ARIES-1 trial (Ambrisentan in Patients With Moderate to Severe Pulmonary Arterial Hypertension), ambrisentan significantly improved the 6MWD in PAH patients, with a MD of 51 m, when the highest dose was compared to the placebo group [55]. Finally, in the SERAPHIN trial (Study With an Endothelin Receptor Antagonist in Pulmonary Arterial Hypertension to Improve Clinical Outcome), macitentan significantly improved the 6MWD, with a MD of 12.5 m compared to the placebo group [56]. Thus, the three ERAs have proven to be useful in the treatment of PAH as all improve the 6MWD, WHO functional class, and different hemodynamic parameters. The absence of trials that have directly compared ERA treatments complicates the efficacy assessments of different treatment regimens. Network metanalyses suggest that ambrisentan might be the most appropriate treatment regarding efficacy and tolerability [57]. However, the safety and efficacy, especially of macitentan, must be evaluated in future studies with PAH patients. The same is true for combination treatment regimens. Table 2 lists the relevant active and recruiting trials that are evaluating ERAs in the treatment of PAH.

Apart from PAH, the endothelin system has been implicated in many other pathologies. More particularly, its involvement in cardiovascular disease has recently be summarized by Barton and Yanagisawa [10]. Moreover, trials with the ET_A_-selective ERA darusentan showed BP reduction in models of salt-sensitive hypertension [58,59] and in patients with resistant hypertension [60,61,62]. Effects of aprocitentan in patients with resistant hypertension are currently under investigation in the PRECISION phase III trial (ClinicalTrials identifier NCT03541174). In addition, darusentan treatment attenuated the progression of experimental atherosclerosis [63]. Preclinical studies further suggest a therapeutic potential of ERA treatment in patients with coronary artery disease or acute coronary syndrome/myocardial infarction [10], as well as in the termination of coronary vasospasm [64]. However, so far, there is no evidence for clinical benefits of ERA therapy for heart failure. Further medical disorders in terms of ERA treatment are discussed in the following sections.

## 5. Cancer

**Figure 3:**
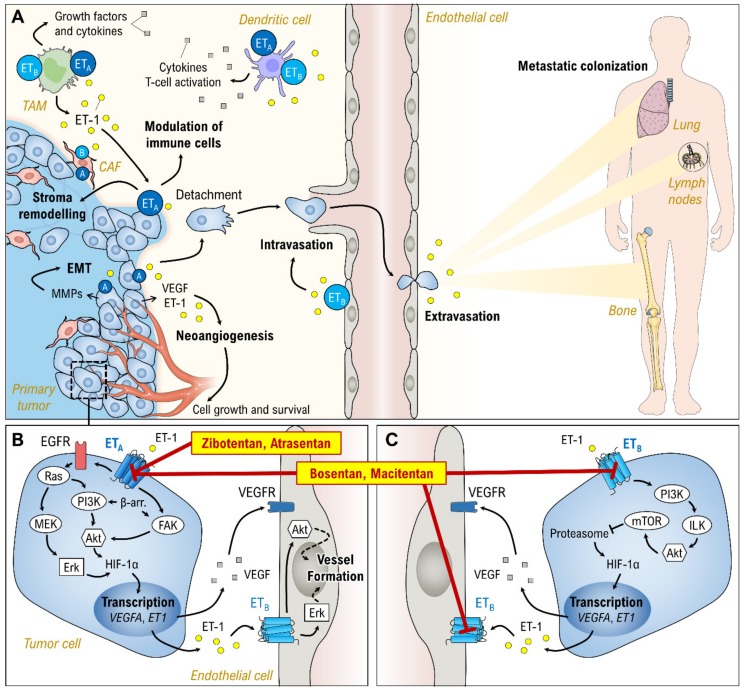
(**A**) Involvement of ET-1 (yellow circles) in cancer progression and metastasis. (**B**) Postulated ET-1 receptor A signaling in tumor cells. Binding of ET-1 to the ET_A_ receptor induces VEGF (gray squares) and ET-1 expression by upregulation of HIF-1α. (**C**) Possible involvement of ET-1 receptor B on the regulation of HIF-1α stability. Arrows describe cause–effect relationships. Red lines indicate receptor antagonism of the respective ERAs. Akt, protein kinase B; β-arr., β-arrestin; CAF, cancer-associated fibroblast; EGFR, epidermal growth factor receptor; EMT, epithelial-mesenchymal transition; Erk, extracellular signal-regulated kinase; FAK, focal adhesion kinase, ILK, integrin-linked kinase; MEK, Raf–mitogen-activated protein kinase (MAPK)/ERK kinase; MMP, matrix metallopeptidases; mTOR, mammalian target of rapamycin PI3K, phosphoinositide 3-kinase; Ras, rat sarcoma proto oncogene; TAM, tumor-associated macrophage; VEGF(R), vascular endothelial growth factor (receptor). Parts of the figure are drawn using pictures from Servier Medical Art (https://smart.servier.com), licensed under a Creative Commons Attribution 3.0 Unported License (https://creativecommons.org/licenses/by/3.0).

ET-1 is a mitogenic and antiapoptotic peptide, and its expression—as well as that of other components of the endothelin axis—is increased in human cancers and has been implicated in their development and progression (Figure 3A) [1,65]. Preclinical animal experiments and cellular models have addressed all components of the endothelin axis in the development and progression of cancer. ET-1 acts as a direct survival and proliferation factor for cancer cells [66] and is involved in the transactivation of other receptors, including the epidermal growth factor receptor (EGFR) [67]. In many tumors, ET-1 exerts a tumor-promoting effect through direct angiogenic effects on endothelial cells and through both autocrine and paracrine pathways in the growing tumor [68,69]. Via binding to the ET_A_ receptor, ET-1 induces vascular endothelial growth factor (VEGF) expression by increasing levels of hypoxia-inducible factor 1α (HIF-1α) (Figure 3B,C) [70,71]. HIF-1α is likely to have a pivotal role in the ‘pro-tumor’ effect of ET-1 and elevated levels of HIF-1α are strongly correlated with metastasis, angiogenesis, cancer resistance and poor prognosis [72]. VEGF stimulates cancer cells and fibroblasts to produce proangiogenic proteases resulting in tumor angiogenesis [73]. In addition, ET-1 directly and indirectly promotes the epithelial-mesenchymal transition (EMT), invasion, migration and metastasis of cancer cells [1,74]. ET-1 also mediates recruitment, proliferation and differentiation of fibroblasts into myofibroblasts associated with tumors [75] and mediates the interaction between cancer cells and immune cells [1]. Taken together, activation of the endothelin receptors likely promotes tumor progression through many different mechanisms, including apoptosis inhibition, matrix remodeling, cell proliferation and activation of osteoblasts that can result in bone deposition in skeletal metastases [76] (Figure 3A). Nevertheless, the involvement of an endothelin signaling network in tumor angiogenesis suggests the use of specific ET_A_ receptor antagonists may be a new therapeutic strategy to improve antitumor treatment by suppressing both tumor cell growth and neovascularization (Figure 3B) [68].

Depending on the relative expression of ET_A_ and ET_B_ receptors present in human cancer (Table 3), ERAs with the right profile might be useful in cancer treatment. Preclinical ERA studies have been conducted on various cancer types with promising results, although it must be emphasized that the concentration of ERAs necessary to impact apoptosis in cancer cells was very high compared to the effective concentrations in cardiovascular disease [1]. There is a link between the autocrine activation of ET_A_ and EGFR leading to EMT, chemoresistance and metastasis in epithelial ovarian cancer cells. A study showed that the ET_A_ antagonist zibotentan combined with an EGFR antagonist greatly reduced the proliferation and invasion of the tumor cells [77]. Macitentan in combination with other drugs, but not as monotherapy, exhibited marked antitumoral effects in an experimental model of multidrug-resistant ovarian tumors [78]. A preclinical study of colon cancer showed that zibotentan reduced cell proliferation. However, cell migration was more inhibited by an ET_B_ antagonist, and a combined antagonist was more effective regarding cell contraction [79].

Potentiation of chemotherapy effects by ERAs were reported for different cancer types [113,114,115,116]. This may be explained by the binding ability of ERAs to membrane transporters, such as P-gp, that are involved in multidrug resistance in several neoplasms [117] and whose overexpression is traditionally linked to poor cancer prognosis [118]. ERAs can reduce P-gp functions at the blood–brain barrier resulting in less transport of chemotherapeutics not only in brain cancer but also in other cerebral disorders [119]. In addition, macitentan combined with chemotherapy has demonstrated anti-cancer stem cell activity [1]. Atrasentan enhanced the radiation-induced inhibition of tumor growth [120].

In summary, preclinical and ex vivo studies suggest that malignant cells are dependent on ET-1 with regard to cell growth and survival. Several ERAs have demonstrated promising effects in the context of experimental cancer, with the potential to control survival of the malignant cells and regulate vascular functions. ERAs have shown antitumor effects in cells of both human and animal origin. These encouraging findings ultimately led to the evaluation of ERAs in human clinical trials. These clinical trials have assessed the value of ERAs, alone or in combination with cytotoxic drugs, in the treatment of many cancer types, including prostate, ovarian, breast, colon, kidney and lung. These studies mainly focused on the two selective ET_A_ antagonists atrasentan and zibotentan [16,121,122]. Unfortunately, the results overall have been very disappointing, with no measurable, statistically significant advantages, even though the drugs were well-tolerated (Table 4) [121,122,123,124].

These results merit a discussion of the future of ERAs in connection with human cancer. What causes the discrepancy between the results from preclinical models and the human clinical trials? This phenomenon might be explained by numerous factors. In the preclinical models, the direct effects of on tumor cells were evaluated. However, cell cultures cannot reflect the in vivo situation of tumor biology. In addition, physiological differences and variations in target homology between animals and humans may lead to translational limitations [137]. Novel compounds are also often tested in cancer patients when the established therapies have failed, and the pattern of expression of endothelin receptors might be complex and uncertain. Moreover, it is possible that dual antagonists are more appropriate than single antagonists in cancer treatment. Cancer-associated fibroblasts and tumor-associated macrophages are mandatory for human tumor progression, and these cells express both ET_A_ and ET_B_ (Figure 3A). These aspects were not considered in the trials.

## 6. Renal Disease

In chronic kidney disease (CKD), an injury to tubular or glomerular cells is followed by progressive dysfunction. Both inflammatory and noninflammatory stress affect the glomerulus, resulting in changes in structure, permeability and functions. CKD treatment mainly comprises inhibiting the renin-angiotensin system, but patients remain at high risk of developing serious cardiovascular complications and end-stage kidney disease. Furthermore, the development of new drugs for treating these conditions has been slow to evolve [138]. However, ERAs represent a new hope regarding diabetic nephropathy.

**Figure 4:**
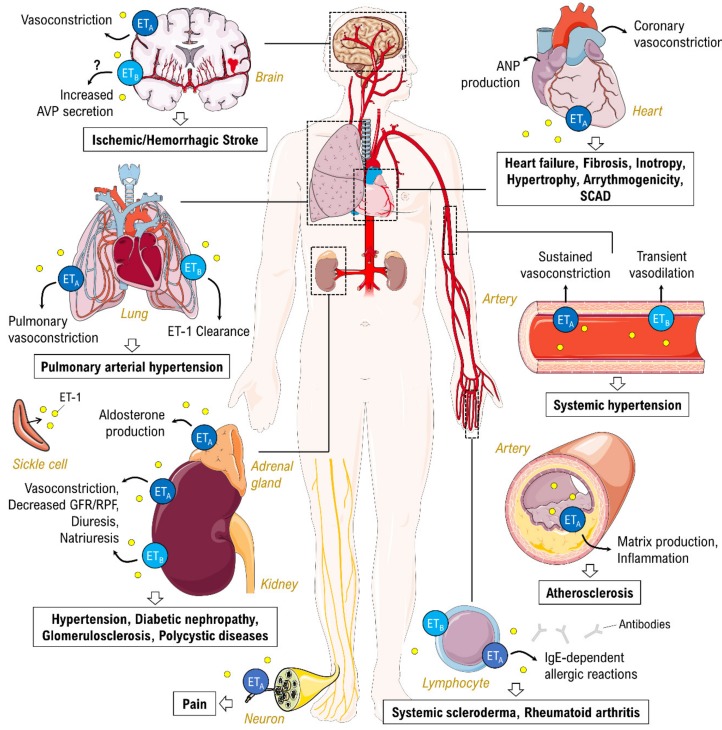
Pathological roles of ET-1 (yellow circles) and endothelin receptor signaling in different diseases. Arrows indicate cause–effect relationships. ANP, atrial natriuretic peptide; AVP, arginine vasopressin; GFR, glomerular filtration rate; RPF, renal plasma flow; SCAD, spontaneous coronary artery dissection; question mark, correlation not exactly known. Parts of the figure are drawn using pictures from Servier Medical Art (https://smart.servier.com), licensed under a Creative Commons Attribution 3.0 Unported License (https://creativecommons.org/licenses/by/3.0).

Within the kidney, ET_A_ activation mediates sodium retention, inflammation and fibrosis, whereas sodium excretion via the NO pathway as well as protection against ET_A_-receptor-induced actions on inflammation and fibrosis is mediated by ET_B_ receptor activation [139] (Figure 4). Early trials investigated patients with cardiovascular and kidney disease and focused on dual ET_A_ and ET_B_ receptor antagonists. Hypothetically, the more potent selective ET_A_ receptor antagonists have a greater potential for benefit, although the risk of AEs might be equally increased. A significant AE for high doses of ET_A_ receptor antagonists is fluid retention, which is potentially life threatening in at-risk patients. Thus, special care is necessary when considering treating these patients with selective ERAs [140]. However, when the low effective doses of the selective ET_A_ antagonist atrasentan were evaluated in an early phase II study, the frequency of fluid retention was similar to that of the placebo group [141].

In 2013, the double-blind, randomized, placebo-controlled SONAR trial (Study Of Diabetic Nephropathy With Atrasentan) was initiated to evaluate the long-term effects of atrasentan treatment in patients with type 2 diabetes and CKD. The trial incorporated a personalized approach into the design, namely by selecting individuals who responded well to atrasentan in a run-in period. Atrasentan significantly reduced the risk of renal events in the selected patients with diabetes and CKD compared to placebo [142], and the results represented one of the first successful trials of therapeutics that target the kidney in diabetes patients in more than 10 years [138]. Moreover, preclinical and small clinical studies have shown that ET_A_ receptor antagonists are effective in models of non-diabetic CKD, including hypertension-induced kidney disease and focal segmental glomerulosclerosis (Table 5) [143]. Consequently, ET_A_ receptor antagonists hold much promise for reducing the disease progression of a variety of renal diseases. Drugs like atrasentan have a great potential. Unfortunately, the future of ET_A_ receptor antagonist treatment in kidney disease is uncertain given that patents already have or soon will expire [138]. Pharmaceutical companies may look towards newer combined agents such as sparsentan, which targets both the ET_A_ receptor and the angiotensin II type 1 receptor [144].

De Zeeuw et al. [147] showed that the ERA atrasentan lowers albuminuria and improved blood pressure and the lipid spectrum in patients with type 2 diabetic nephropathy receiving RAS inhibitors. However, the clinical utility of ERA may be limited by fluid retention [148]. A post hoc analysis of the phase IIb atrasentan trials assessing albuminuria reduction in 211 patients with type 2 diabetes showed that the ERA-associated fluid retention occurred mainly in patients with diabetes and nephropathy with a lower estimated glomerular filtration rate or who received a higher dose of atrasentan. Moreover, the albuminuria-reducing efficacy of atrasentan is not impaired by fluid retention [148]. Lin et al. [146] demonstrated the results from three phase II trials with 257 participants (NCT01356849, NCT01399580, and NCT01424319). No significant association between atrasentan exposure and peripheral edema was found (doses: 0.5, 0.75, 1.25 mg). The rates of peripheral edema were comparable in patients receiving active treatment and placebo [146].

Earlier results proposed that ET_A_ antagonism is reducing circulating lipids [161]. In a secondary analysis of a crossover study (NCT00810732), 27 participants with predialysis CKD treated with an optimal cardio- and renoprotective therapy were randomly assigned for treatment with a six-week dosing with placebo, sitaxentan, or long-acting nifedipine. The clinical trial showed that selective ET_A_ antagonism improves lipid profiles in optimally managed CKD patients [161]. ET_A_ receptor blockade may be a novel strategy to reduce cardiovascular disease risk in CKD [161]. In addition, the decrease in proteinuria and the lowered blood pressure following sitaxentan therapy were associated with increases in urine ET-1/creatinine. The reduction in pulse-wave velocity was associated with a decrease in ADMA. Therefore, ET_A_ receptor antagonism may modify risk factors for cardiovascular disease in CKD [145].

In sickle-cell disease (SCD) erythrocytes stimulate endothelial ET-1 production [162]. Patients suffer from tissue damage and life-threatening complications due to vaso-occlusive crisis that affects the kidney and other organs. Thus, the excretion of ET-1 with the urine reflects the degree of renal injury [163]. Treatment with bosentan [164] or ambrisentan [165] could reduce organ injury and mortality in an SCD mouse model. Currently, an ongoing phase I trial (NCT02712346) is investigating the effects of ambrisentan on renal function, proteinuria, and macro-/microvascular function. The first results are expected soon.

## 7. Fibrotic Diseases

Fibrosis is defined by the overgrowth, scarring, and hardening of tissues occurring in response to tissue injury, when mainly myofibroblasts are activated followed by the replacement of normal tissue with scar tissue [166]. Increased fibroblast proliferation and accumulation of extracellular matrix (ECM) proteins are characteristic for fibrosis. Interestingly, same effects are caused by ET-1-mediated activation of ET_A_ and ET_B_ on fibroblasts [167]. This activation is a key factor in the pathogenesis of fibrosis and may be an important mediator of other profibrotic effects [168,169]. ET-1 increases the ECM production (collagen types I/III, fibronectin) [170,171,172] and inhibits the activity of enzymes responsible for ECM degradation (collagenase) [173]. Furthermore, it interacts with MEK/ERK, transforming growth factor β and nuclear factor ‘kappa-light-chain-enhancer’ of activated B-cells (NF-κB) signaling [168,174] inducing profibrotic effects. Clinical evidence for the involvement of ET-1 in fibrosis was first reported in scleroderma patients (see Section 9).

ERAs may have therapeutic potential in preventing the development of fibrotic diseases. Bosentan were reported to inhibit ET-1-induced fibroblast proliferation and ECM deposition. Furthermore, it reduced cardiac, pulmonary, hepatic and renal fibrosis in different disease models characterized by the activation of the endothelin axis [168]. In addition, bosentan could reverse existing fibrosis, possibly by stimulating collagenase expression. Inhibition of endothelin signaling with ERAs also reduced hepatic fibrogenic response in experimental models of liver disease [175,176].

According to immunohistochemistry performed on human lung tissue and experiments in animal models, the endothelin system might also be involved in the pathogenesis of pulmonary fibrosis [168,169,177,178]. Currently, there is no curable treatment for this disease, and approved compounds such as the tyrosine kinase inhibitor nintedanib can only slow down progression [179]. The endothelin axis was shown activated in the pathogenesis in an experimental rodent model of idiopathic pulmonary fibrosis and treatment with bosentan indicated a decrease in pulmonary fibrosis in one preclinical study [180]. Bosentan, ambrisentan, and macitentan have been evaluated in clinical trials on patients with pulmonary fibrosis (Table 5) [150,151,152,153,181]. Unfortunately, all have produced negative or neutral results. One trial was prematurely stopped given an increased disease progression in the treated arm [152].

## 8. Cerebral Vasospasm

Vasospasm is the narrowing of arteries (vasoconstriction) after a subarachnoid hemorrhage (SAH). Endothelin is considered to be a key mediator of vasospasm following a SAH. After a SAH in animals and humans, most studies report that there is an increase in endothelin in the blood and cerebrospinal fluid [182]. The SAH pathogenesis is unclear, but it is widely accepted that the interaction between ET-1 and NO is critical for maintaining adequate cerebral vascular dilatation and sufficient cerebral blood flow during a SAH. NO is a potent endogenous vasodilator; it directly acts on VSMCs to cause vascular relaxation. Studies have shown that the administration of ET-1 antagonists or inhibitors of ECE—which is responsible for activating endothelin—can prevent a vasospasm [183]. The synthetic ET_A_ receptor antagonist clazosentan is often considered the most promising drug that has been studied for the prevention or reversal of cerebral vasospasm [183]. It decreases and reverses a cerebral vasospasm after an experimental SAH in animal models [184]. Twenty published reports demonstrated that endothelin antagonists prevented or reduced a vasospasm after an experimental SAH in rats, rabbits, dogs and monkeys [185]. The double-blind, randomized CONSCIOUS-1 trial (Clazosentan to Overcome Neurological Ischemia and Infarction Occurring After Subarachnoid Hemorrhage) studied the effects of clazosentan in cerebral vasospasm. The drug decreased the incidence of severe vasospasm and delayed ischemic neurological deficits and new infarcts seen on computed tomography scans in a dose-dependent manner [154]. A subgroup analysis of patients participated in the terminated CONCIOUS-2 and CONCIOUS-3 phase III trials indicated a dose-dependent reduction in vasospasm-related morbidity, but no significant effect on overall survival [186].

## 9. Systemic Scleroderma

Systemic scleroderma or systemic sclerosis (SSc) is a rare and complex autoimmune disease characterized by fibrosis of the skin and internal organs and widespread vasculopathy. Raynaud’s phenomenon is often the first manifestation of SSc. Vasospasm of the digital arteries leads to the three characteristic phases of pallor, cyanosis and erythema, all of which lead to reduced blood flow, total loss of oxygen supply and reperfusion. Recurrent episodes of an ischemia-reperfusion injury and the subsequent generation of reactive oxygen species can result in ischemic damage and necrotic lesions called digital ulcers [187].

In SSc patients and patients with Raynaud’s phenomenon, plasma ET-1 concentrations are elevated and correlate with the severity of the disease [187,188,189,190]. In addition to its role as a biomarker of vascular disease, ET-1 itself might contribute to the fibrotic and vasculopathic aspects of SSc because it has been shown to stimulate fibroblast and smooth muscle cell proliferation as well as proinflammatory effects [191,192]. In addition, SSc patients develop autoantibodies directed against ET_A_ predicting the occurrence of new digital ulcers [193]. Treatment with macitentan did not reduce the development of new digital ulcers in SSc patients [157]. In contrast, a reduction of up to 48% in the number of new digital ulcers was found after bosentan therapy, which affects the development but not the progression of existing digital ulcers [159].

Patients suffering from autoimmune diseases such as SSc often develop PAH that may require ERA treatment [194]. Bosentan, sitaxsentan, and ambrisentan have been demonstrated to be effective in the treatment PAH related to scleroderma (SSc-PAH) [158,195]. This effect might be mediated through a vasodilatory and antifibrotic effect, thus making these agents attractive as potential disease modifying agents for SSc [196].

## 10. Pain Management

The management of chronic pain is a worldwide challenge, and current pain therapies are often ineffective and have considerable side effects. Endothelin and its receptors are present in the pain signaling pathway. Dependent on its local concentration, ET-1 itself can have both nociceptive or antinociceptive properties [197,198]. ET-1 potentiates the effects of an algogen, such as capsaicin and arachidonic acid, as well as directly activating nociceptors [199]. Moreover, ET-1 is involved in conditions such as diabetic neuropathy, cancer pain, neuropathic pain and inflammatory pain [200,201]. An elevation in plasma ET-1 has been measured in animal models of several pathological conditions where pain is an important symptom. These include Raynaud’s disease, cancer (e.g., prostate cancer), complex pain syndrome, vaso-occlusive crisis, and acute chest syndrome associated with sickle cell disease [200].

Many human diseases are potential candidates for ET_A_ receptor antagonist pain reduction therapies, and ERAs have shown promising results in this regard. Most trials with ERAs for the treatment of pain have been conducted in animals, including studies on cancer pain [202] and sickle cell disease [203]. The current knowledge suggests that the ET_A_ receptor is a potential target for managing pain associated with these diseases. In a small observational study, three patients with secondary Raynaud’s phenomenon who received bosentan for 16 weeks experienced measurably decreased pain severity as well as improved peripheral thermoregulation [204]. A reduction of pain was also reported in trials of prostate and bone cancer pain patients [121]. Although several clinical trials with ERAs have been completed, most have focused on actions of the endothelin axis other than pain relief. Therefore, it is difficult to gain the maximum benefit from these trials. Furthermore, the full-scale effects of ET-1 on nociception need to be understood in more detail and evaluated thoroughly in the future.

Opioids are highly effective drugs for treating pain. Highly potent opioid receptor agonists have been developed. However, the drugs are known to be very addictive, and tolerance as well as various AEs of these drugs remain a problem [205]. Opioid actions are mediated through the activation of G-protein-coupled receptors, which activate and regulate various second messenger pathways. Chronic exposure to opioids leads to a spectrum of cellular adaptations resulting in the downregulation of mechanisms and desensitization. Thus, in some addicts and chronic pain patients, a 100-fold increase over the normal analgesic morphine dose has been reported to produce only minor physiological effects [205]. Animal studies have shown that endothelin may modulate the pharmacological effects of morphine, and that opioids and endothelin may interact [206,207]. Moreover, other animal studies have provided evidence that ET_A_ receptor antagonists can potentiate the analgesic response induced by morphine [208], and that they can promote coupling of G-protein to its receptors in morphine-tolerant mice, where an uncoupling of G-proteins occurs [209]. In addition, ET_A_-selective ERAs were reported to increase the secretion of β-endorphin and Leu-enkephalin [210].

It has been hypothesized that ET_A_ receptor antagonists might minimize the withdrawal symptoms of opioids following the development of tolerance and dependence. This hypothesis has been investigated in animal studies. A selective ET_A_ receptor antagonist was effective in reducing several opioid withdrawal symptoms in mice. These data suggest that the endothelin receptors are involved in CNS pathways in opioid withdrawal [211]. Consequently, ET_A_ receptor antagonists with the right pharmacokinetic properties might be beneficial in combination therapy with opioids in the future, regarding both opioid tolerance and withdrawal.

## 11. Conclusions

The ERAs bosentan, ambrisentan, and macitentan target the endothelin pathway in different ways and have proven useful in the treatment of PAH. All compounds improve the 6MWD, WHO functional class, and various hemodynamic parameters contributing to relieve symptoms and slower disease progression. Due to the pleiotropic effects of endothelin, ERAs have been evaluated in connection with numerous other diseases. Although the results have generally been inconsistent, several studies have produced promising results. Selective ERAs, including bosentan, have demonstrated to be effective in SSc-PAH, and they could potentially act as disease modifying agents or help decrease the development of vasospasm-related digital ulcers. Similarly, the selective ERA clazosentan has been shown to be effective in preventing severe cerebral vasospasm and delaying ischemic neurological deficits as well as new cerebral infarcts.

Some of the most promising recent results regarding ERA treatment have involved CKD. In addition, ERAs represent a new treatment strategy for diabetic nephropathy. The selective ERA atrasentan significantly reduced the risk of renal events in patients with diabetes and CKD. Furthermore, preclinical and small clinical studies have shown that ET_A_ receptor antagonists are effective in various models of non-diabetic CKD, making ET_A_ receptor antagonists promising agents, that could potentially slow down disease progression of a variety of renal diseases.

Preclinical studies suggest that malignant cells may be dependent on ET-1 concerning cell growth and survival, and several ERAs have demonstrated promising effects, including the potential to control survival of the malignant, but clinical trials with ERAs in the treatment of different types of cancer have not been able to produce measurable statistically significant positive results. The same is true for studies that have evaluated the use of ERAs in the treatment of cardiovascular and fibrotic diseases. The field of ET-1 cancer therapeutics will likely be transformed over the years to come, especially facilitated by the growing knowledge of the genomic features of tumor cells and microenvironments and noninvasively biomarker detection in early stages of cancer. The use of deeply developing patient-derived models could be applicable and might comprise a new opportunity in ET-1 therapeutic development [124]. ERAs could prove beneficial in pain management, and the full-scale effects on nociception and opioid tolerance is yet to be evaluated in detail. A better knowledge of the pathophysiology of pain in the future could contribute hereto.

## 12. Outlook

ERAs remain an important part of PAH treatment, but the use of the drugs may not be limited to this condition. Promising results regarding the use of these drugs in other diseases have been produced. However, more studies are needed to assess the benefits and safety of ERA treatment in various, well defined patient groups, both with known and new ERAs. Additional studies are needed to shed light on the efficacy of different combination therapy regimens.

The interesting, unique and positive results regarding treatment of diabetic nephropathy—and a rare successful trial of therapeutics that target the kidney in patients with diabetes—have been partially overshadowed by the uncertain future of ERA treatment due to patent expiration. Furthermore, many (older) trials with ERAs have failed to produce any positive results due to non-optimal patient selection and overdosing [212]. Despite the pleiotropic effects of endothelin, it seems that the drugs have no significant effects in the treatment of cardiovascular and fibrotic diseases. Nevertheless, ERAs could potentially play an important role in the treatment of a variety of other diseases in the future. Cancer scientists are working on a novel strategy to increase the blood flow to the tumor by using the ET_B_ receptor agonist SPI-1620 (IRL-1620) in order to improve drug delivery or to augment the effects of radiation therapy [134,213]. Most recently, a nanobody was constructed that may be useful as a potential new ET_B_ antagonist in the treatment of melanoma [214]. Further studies suggested beneficial effects of ERA treatment on cognitive function in experimental models of vascular dementia [215,216]. The therapeutic potential of ERAs in vascular dementia or in Alzheimer disease has not yet been studied in humans yet, but preclinical and pathology data offer hope. With focus on the pro-inflammatory effects of ET-1, ERAs may also reveal therapeutic benefit for other autoimmune diseases than SSc, such as rheumatoid arthritis or lupus erythematosus.

Using the lessons we have learned, it should be possible to design and conduct successful trials reflecting the full therapeutic potential of ERAs.

## Figures and Tables

**Table 1:** A comparison of the pharmacokinetic characteristics and adverse events of ERAs.

	Bosentan [20]	Ambrisentan [21]	Macitentan [22]	Sitaxsentan [23]	Atrasentan [24]	Clazosentan [17]	Zibotentan [25]	Aprocitentan [26]
Approval status	FDA: 2001 EMA: 2002	FDA: 2007 EMA: 2008	FDA: 2013 EMA: 2013	Withdrawn	Experimental	Experimental	Experimental	Experimental
Receptor selectivity	Non-selective	Selective (ET_A_)	Non-selective	Selective (ET_A_)	Selective (ET_A_)	Selective (ET_A_)	Selective (ET_A_)	Non-selective
Administration and dose recommendation	Oral twice daily (62.5–125 mg)	Oral once daily (5–10 mg)	Oral once daily (10 mg)	Oral once daily (i.e., 100 mg)	Oral once daily (i.e., 40 mg)	Intravenous (30–60 mg/h for 6 h)	Oral once daily (10–100 mg [19])	Well tolerated ≤100 mg/d for 10 days.
Oral bio-availability (%)	49.8 [27]	80	74 [28]	>90	N.R.	N.R.	N.R.	Oral active
T_½_ (h)	5	15	16	~10	24	1–2	~8	N.R.
T_max_ (h)	3	2	8	1–2	1.5	N.R.	1	N.R.
Metabolizing enzymes	CYP3A4 and CYP2C9	CYP3A4, CYP2C19, UGTs 1A9S, 1A3S and 2B7S	CYP3A4, CYP2C19	CYP2C9 [29]	Glucoronidation, CYP3A oxidation	CYP2C9 [30]	CYP3A4	CYP3A4, CYP2C19
Elimination pathway	Biliary excretion, bio-transformation	Biliary excretion, bio-transformation	Renal, fecal, bio-transformation	Renal, fecal	N.R.	Fecal, biliary excretion	Renal	Renal, fecal, bio-transformation
Common adverse effects	Headache, nausea, vomiting, flushing, palpitations	Peripheral edema, nasal congestion, headache, dizziness	Naso-pharyngitis, headache	Nausea, headache, peripheral edema, flushing	Headache, rhinitis, asthenia, peripheral edema	Headache, nausea, nasal obstruction, vomiting	Headache, nausea	Hypertension, headache, nasopharyngitis, decrease in hemoglobin and hematocrit
Serious adverse effects	Anemia, abnormal hepatic function	None known	Anemia	Serious impact on liver function	Hypotension, hyponatremia [31]	None known	Lesions of olfactory epithelium [32]	N.R.

CYP, cytochrome P450; EMA, European Medicines Agency; FDA, Food and Drug Administration; N.R., not reported; UGT, Uridine 5′5′-diphosphate glucuronosyltransferase.

**Table 2:** Recruiting and active trials on the use of ERAs in the treatment of PAH.

Trial	Design	Objective	Study Size	Status
NCT01827059: A randomized placebo-controlled trial to analyze changes in pulmonary arterial pressures at peak exercise in congenital heart disease patients with exercise-induced PAH before and after treatment with bosentan, compared to placebo (BICYCLE)	Interventional, Randomized, Parallel assignment, Double-blind, Phase II	The objective of this trial is to analyse changes in pulmonary arterial pressure at peak exercise before and after treatment with bosentan, compared to placebo, in patients with con-genital heart disease.	12 participants	Unknown
NCT01347216: Prospective registry of newly initiated therapies for pulmonary hypertension (COMPERA)	Observational, Cohort, Prospective	The aim of this trial is to compare the results of the manifold options for mono- and combination therapy in the treatment of PAH.	10,000 participants	Recruiting
NCT01406327: Drug use investigation for VOLIBRIS^®^ (ambrisentan) (PAH)	Observational	The goal of this trial is to evaluate the incidence of adverse events in Japanese PAH patients treated with ambrisentan.	900 participants	Active, not recruiting
NCT03809156: Upfront riociguat and ambrisentan combination therapy for PAH: a safety and efficacy pilot study	Interventional, Single group assignment, No masking, Phase IV	The aim of this trial is to evaluate the efficacy and safety of first-line combination therapy using ambrisentan and riociguat in patients with PAH.	20 participants	Recruiting
NCT01342952: An open-label, long-term extension study for treatment of pulmonary arterial hypertension in pediatric patients aged 8 years up to 18 years who have participated in AMB112529 and in whom continued treatment with ambrisentan is desired	Interventional, Single group assignment, No masking, Phase II	The primary objective of this trial is to assess the long-term tolerability and safety of ambrisentan in a pediatric PAH population. Secondary objectives include all-cause mortality and change from baseline on efficacy parameters in Study AMB112529.	66 participants	Recruiting
NCT02932410: A multicenter, open-label, randomized, event-driven study to assess efficacy, safety and pharmacokinetics of macitentan versus standard of care in children with PAH (TOMORROW)	Interventional, Randomized Parallel assignment, No masking, Phase III	The goal of this trial is to evaluate the efficacy, safety and pharmacokinetics of macitentan in children with PAH.	300 participants	Recruiting
NCT03422328: Multicenter, single-arm, open-label, long-term safety study with macitentan in patients with pulmonary arterial hypertension previously treated with macitentan in clinical studies (UMBRELLA)	Interventional, Single group assignment no masking, Phase III	The aim of this trial is to evaluate the long-term safety of mac-itentan, and to provide continued treatment with macitentan in patients with PAH who were treated with macitentan in previous clinical studies.	94 participants	Enrolling by invitation
NCT00667823: Long-term single-arm open-label extension study of the SERAPHIN study, to assess the safety and tolerability of ACT 064992 in patients with symptomatic PAH (SERAPHIN OL)	Interventional, single group assignment, no masking, Phase III	The objective of this trial is to evaluate the long-term tolerability and safety of maci-tentan in patients with symptomatic PAH.	550 participants	Active, not recruiting
NCT02126943: US-based, observational, drug registry of opsumit^®^ (macitentan) new users in clinical practice (OPUS)	Observational, Cohort, Prospective	The goal of this trial is to assess safety and to describe outcomes and clinical characteristics of patients newly treated with macitentan.	5000 participants	Recruiting
NCT03904693: Prospective, multi-center, double-blind, randomized, active-controlled, triple-dummy, parallel-group, group-sequential, adaptive phase 3 clinical study to compare the efficacy and safety of macitentan and tadalafil monotherapies with the corresponding fixed dose combination in subjects with PAH, followed by an open-label treatment period with macitentan and tadalafil fixed dose combination therapy	Interventional, Randomized, Parallel assignment, Double blind, Phase III	The aim of this trial is to assess the benefits of a fixed-dose combination therapy with macitentan and the phosphodiesterase type 5 inhibitor tadalafil compared to monotherapy with macitentan or tadalafil.	170 participants	Recruiting
NCT03362047: ’Untersuchung des Einflusses PAH-spezifischer Medikation auf die rechtsventrikuläre Funktion bei Patienten mit PAH unter basalen Bedingungen’	Interventional, Randomized, Parallel assignment, no masking, Phase II	The objective of this pilot study is to determine the therapeutic effect of two parallel groups treated with either macitentan or the soluble guanylate cyclase (sGC) stimulator riociguat.	30 participants	Recruiting

**Table 3:** Expression of ET-1 receptors in human cancer.

Tumor Type	Endothelin Receptors	Effects Associated with Endothelin Receptor Expression	References
Bladder cancer	ET_A_, ET_B_	Reduced survival	[80,81]
Breast cancer	ET_A_	Reduced survival; increased invasion; bone metastasis	[82,83]
Cervical cancer	ET_A_	N.R.	[84]
Colorectal cancer	ET_A_	Reduced survival; increased tumor grade	[85,86]
Gastric cancer	ET_A_, loss of ET_B_	N.R.	[87,88]
Glioblastoma	ET_A_, ET_B_	N.R.	[89,90,91]
Head and neck cancer	ET_A_	Reduced survival	[92,93]
Hepatocellular carcinoma	ET_A_, loss of ET_B_	Cell migration; invasion	[94,95]
Lung cancer	NSCLC: ET_A_ SCLC: ET_B_	NSCLC (adenocarcinoma): Reduced survival	[96,97]
Malignant melanoma	ET_B_	Aggressive phenotype; cancer progression; metastasis to lymph nodes	[98,99]
Ovarian cancer	ET_A_	Increased tumor grade; chemoresistance; metastasis	[100,101]
Pancreatic cancer	ET_A_, ET_B_	Proliferation; angiogenesis	[102,103,104]
Prostate cancer	ET_A_	Increased tumor grade; bone metastasis	[105,106]
Renal cell carcinoma	ET_A_, ET_B_	Reduced survival (ET_B_), increased tumor grade (ET_A_)	[107,108,109,110]
Thyroid cancer	ET_A_	PTC: Tumor growth; lymph node metastases	[111]
Vulvar cancer	ET_B_	Reduced survival	[112]

N.R., not reported; NSCLC, non-small-cell lung cancer; PTC, papillary thyroid cancer; SCLC, small-cell lung cancer.

**Table 4:** ERAs in cancer therapy: Results of clinical trials.

ERA + Combination	Receptor Antagonism	Tumor Type	Clinical Development	Results	Ref.
Zibotentan	ET_A_	Prostate cancer (non-metastatic CRPC)	Phase III	No significant effect.	[125]
		Prostate cancer (metastatic CRPC)	Window Study, NCT01168141	N.R.	
		Prostate cancer (non-metastatic HRPC)	Phase III, NCT00626548	Study terminated.	
		Prostate cancer (metastatic HRPC)	Phase III, NCT00554229	No significant effect on OS.	[122]
+Docetaxel			Phase III, NCT00617669	No significant effect on OS.	[126]
+Pemetrexed		Lung cancer (NSCLC)	Phase II	No survival or progression advantage.	[127]
Atrasentan	ET_A_	Prostate cancer (non-metastatic HRPC)	Phase III	No significant effect.	[128]
		Prostate cancer (metastatic HRPC)	Phase III	No delay in disease progression.	[129]
+Docetaxel +Prednisone		Prostate cancer (metastatic CRPC)	Phase III, NCT00134056	No effect on OS or PFS; patients with highly elevated markers of bone turnover may benefit from atrasentan.	[130,131,132]
		Metastatic renal cell carcinoma	Phase II	No significant effect.	[133]
YM598	ET_A_	Prostate cancer	Phase II, NCT00050297	N.R.	
		Metastatic prostate cancer	Phase II, NCT00048659	N.R.	
SPI-1620 +Docetaxel	ET_B_	Biliary Cancer	Phase II, NCT01773785	Study terminated.	[134]
Bosentan	ET_A_, ET_B_	Metastatic melanoma	Phase II,	Possible benefit in disease stabilization.	[135]
+Dacarbazine			Phase II, NCT01009177	No effect on tumor progression.	[136]
+Nab-paclitaxel +Gemcitabine		Pancreatic cancer	Phase I, NCT04158635	Not yet recruiting.	
Macitentan + Temozolomide	ET_A_, ET_B_	Newly diagnosed glioblastoma	Phase I, NCT02254954	Study terminated.	
		Recurrent glioblastoma	Phase I, NCT01499251	Study terminated.	

CRPC, castration-resistant prostate cancer; HRPC, hormone-refractory prostate cancer; N.R., not reported; NSCLC, non-small-cell lung cancer; OS, overall survival; PFS, progression-free survival.

**Table 5:** ERAs in therapy of various diseases beyond PAH, cardiovascular disease and cancer.

Disease	ERA	Receptor Antagonism	Clinical Development	Results	Ref.
Chronic kidney disease	Sitaxsentan	ET_A_	Phase II, NCT00810732	Reduced GFR, proteinuria and BP.	[143,145]
	BQ-123	ET_A_	Phase I, NCT00722215	N.R.	
Diabetic nephropathy	Avosentan	ET_A_	Phase III, NCT00120328	Study terminated.	
	Atrasentan	ET_A_	Phase II, NCT01399580	Reduced urinary albumin/creatinine ratio.	[146]
			Phase II, NCT01356849		
			Phase II, NCT01424319	Reduced urine albumin/creatinine ratios and albuminuria.	[146,147,148]
			Phase II, NCT00920764	Preserved estimated GFR.	[149]
Focal segmental glomerulosclerosis	Sparsentan	ET_A_ (+ARB)	Phase II, NCT01613118	Reduced proteinuria.	[144]
Sickle cell disease	Ambrisentan	ET_A_	Phase I, NCT02712346	Not yet reported.	
Idiopathic pulmonary fibrosis	Bosentan	ET_A_, ET_B_	Phase III, NCT00391443	Well tolerated; no significant effect.	[150]
			Phase II, NCT00071461	No effect on 6MWD; little improvement in QOL	[151]
	Ambrisentan	ET_A_	Phase III, NCT00768300	No significant effect.	[152]
	Macitentan	ET_A_, ET_B_	Phase II, NCT00903331	No significant effect.	[153]
Malignant hypertension	Aprocitentan	ET_A_, ET_B_	Phase III, NCT03541174	Not yet reported.	
Cerebral vasospasm	Clazosentan	ET_A_	Phase II, NCT00111085	Decreased the incidence of severe vasospasm.	[154]
			Phase III, NCT00558311	No significant effect.	[155]
			Phase III, NCT00940095	Reduced mobidity; no dose improved outcome.	[156]
Systemic sclerosis (digital ulcers)	Macitentan	ET_A_, ET_B_	Phase III, NCT01474109	No significant effect.	[157]
			Phase III, NCT 01474122	Study terminated.	
	Bosentan	ET_A_, ET_B_	RAPIDS-1	Improvement in hand function.	[158]
			RAPIDS-2	Reduced occurrence of new digital ulcers; no effect on healing.	[159]
			Prospective observational study	Improvement of functionality and QOL by reducing disease-related symptoms.	[160]

ARB, angiotensin II type 1 receptor antagonist; BP, blood pressure; GFR, glomerular filtration rate; N.R., not reported, QOL, quality of life.

## References

[1] Aubert J.D., Juillerat-Jeanneret L. (2016). Endothelin-Receptor Antagonists beyond Pulmonary Arterial Hypertension: Cancer and Fibrosis. J. Med. Chem..

[2] Macarthur H., Warner T.D., Wood E.G., Corder R., Vane J.R. (1994). Endothelin-1 Release from Endothelial Cells in Culture Is Elevated Both Acutely and Chronically by Short Periods of Mechanical Stretch. Biochem. Biophys. Res. Commun..

[3] Malek A., Izumo S. (1992). Physiological fluid shear stress causes downregulation of endothelin-1 mRNA in bovine aortic endothelium. Am. J. Physiol. Cell Physiol..

[4] Wesson D.E., Simoni J., Green D.F. (1998). Reduced extracellular pH increases endothelin-1 secretion by human renal microvascular endothelial cells. J. Clin. Investig..

[5] Matsuura A., Yamochi W., Hirata K., Kawashima S., Yokoyama M. (1998). Stimulatory interaction between vascular endothelial growth factor and endothelin-1 on each gene expression. Hypertension.

[6] Peifley K.A., Winkles J.A. (1998). Angiotensin II and Endothelin-1 Increase Fibroblast Growth Factor-2 mRNA Expression in Vascular Smooth Muscle Cells. Biochem. Biophys. Res. Commun..

[7] Aro A., Teirilä J., Gref C.-G. (1990). Dose-dependent effect on serum cholesterol and apoprotein B concentrations by consumption of boiled, non-filtered coffee. Atherosclerosis.

[8] Yang Z., Krasnici N., Luscher T.F. (1999). Endothelin-1 potentiates human smooth muscle cell growth to PDGF: Effects of ETA and ETB receptor blockade. Circulation.

[9] Davenport A.P., Hyndman K.A., Dhaun N., Southan C., Kohan D.E., Pollock J.S., Pollock D.M., Webb D.J., Maguire J.J. (2016). Endothelin. Pharmacol. Rev..

[10] Barton M., Yanagisawa M. (2019). Endothelin: 30 Years From Discovery to Therapy. Hypertension.

[11] Shemyakin A., Salehzadeh F., Bohm F., Al-Khalili L., Gonon A., Wagner H., Efendic S., Krook A., Pernow J. (2010). Regulation of glucose uptake by endothelin-1 in human skeletal muscle in vivo and in vitro. J. Clin. Endocrinol. Metab..

[12] Kohan D.E., Rossi N.F., Inscho E.W., Pollock D.M. (2011). Regulation of blood pressure and salt homeostasis by endothelin. Physiol. Rev..

[13] Houde M., Desbiens L., D’Orleans-Juste P. (2016). Endothelin-1: Biosynthesis, Signaling and Vasoreactivity. Adv. Pharmacol..

[14] Nasser S.A., El-Mas M.M. (2014). Endothelin ETA receptor antagonism in cardiovascular disease. Eur. J. Pharmacol..

[15] Barst R.J., Langleben D., Frost A., Horn E.M., Oudiz R., Shapiro S., McLaughlin V., Hill N., Tapson V.F., Robbins I.M. (2004). Sitaxsentan therapy for pulmonary arterial hypertension. Am. J. Respir. Crit. Care Med..

[16] Jimeno A., Carducci M. (2005). Atrasentan: A novel and rationally designed therapeutic alternative in the management of cancer. Expert Rev. Anticancer Ther..

[17] Van Giersbergen P.L., Dingemanse J. (2007). Tolerability, pharmacokinetics, and pharmacodynamics of clazosentan, a parenteral endothelin receptor antagonist. Eur. J. Clin. Pharmacol..

[18] Morris C.D., Rose A., Curwen J., Hughes A.M., Wilson D.J., Webb D.J. (2005). Specific inhibition of the endothelin A receptor with ZD4054: Clinical and pre-clinical evidence. Br. J. Cancer.

[19] Tomkinson H., Kemp J., Oliver S., Swaisland H., Taboada M., Morris T. (2011). Pharmacokinetics and tolerability of zibotentan (ZD4054) in subjects with hepatic or renal impairment: Two open-label comparative studies. BMC Clin. Pharmacol..

[20] Dingemanse J., van Giersbergen P.L. (2004). Clinical pharmacology of bosentan, a dual endothelin receptor antagonist. Clin. Pharmacokinet..

[21] Buckley M.S., Wicks L.M., Staib R.L., Kirejczyk A.K., Varker A.S., Gibson J.J., Feldman J.P. (2011). Pharmacokinetic evaluation of ambrisentan. Expert Opin. Drug Metab. Toxicol..

[22] Clozel M., Maresta A., Humbert M. (2013). Endothelin receptor antagonists. Handb. Exp. Pharmacol..

[23] Benedict N.J. (2007). Sitaxsentan in the management of pulmonary arterial hypertension. Am. J. Health Syst. Pharm..

[24] Carducci M.A., Nelson J.B., Bowling M.K., Rogers T., Eisenberger M.A., Sinibaldi V., Donehower R., Leahy T.L., Carr R.A., Isaacson J.D. (2002). Atrasentan, an endothelin-receptor antagonist for refractory adenocarcinomas: Safety and pharmacokinetics. J. Clin. Oncol..

[25] Clarkson-Jones J.A., Kenyon A.S., Kemp J., Lenz E.M., Oliver S.D., Swaisland H. (2012). Disposition and metabolism of the specific endothelin A receptor antagonist zibotentan (ZD4054) in healthy volunteers. Xenobiotica.

[26] Sidharta P.N., Melchior M., Kankam M.K., Dingemanse J. (2019). Single- and multiple-dose tolerability, safety, pharmacokinetics, and pharmacodynamics of the dual endothelin receptor antagonist aprocitentan in healthy adult and elderly subjects. Drug Des. Dev. Ther..

[27] Weber C., Schmitt R., Birnboeck H., Hopfgartner G., van Marle S.P., Peeters P.A., Jonkman J.H., Jones C.R. (1996). Pharmacokinetics and pharmacodynamics of the endothelin-receptor antagonist bosentan in healthy human subjects. Clin. Pharmacol. Ther..

[28] Sidharta P.N., Krahenbuhl S., Dingemanse J. (2015). Pharmacokinetic and pharmacodynamic evaluation of macitentan, a novel endothelin receptor antagonist for the treatment of pulmonary arterial hypertension. Expert Opin. Drug Metab. Toxicol..

[29] Dhaun N., Melville V., Kramer W., Stavros F., Coyne T., Swan S., Goddard J., Webb D.J. (2007). The pharmacokinetic profile of sitaxsentan, a selective endothelin receptor antagonist, in varying degrees of renal impairment. Br. J. Clin. Pharmacol..

[30] Van Giersbergen P.L., Dingemanse J. (2007). Effect of gender on the tolerability, safety and pharmacokinetics of clazosentan following long-term infusion. Clin. Drug Investig..

[31] Ryan C.W., Vogelzang N.J., Vokes E.E., Kindler H.L., Undevia S.D., Humerickhouse R., Andre A.K., Wang Q., Carr R.A., Ratain M.J. (2004). Dose-ranging study of the safety and pharmacokinetics of atrasentan in patients with refractory malignancies. Clin. Cancer Res..

[32] Esvelt M.A., Freeman Z.T., Pearson A.T., Harkema J.R., Clines G.T., Clines K.L., Dyson M.C., Hoenerhoff M.J. (2019). The Endothelin-A Receptor Antagonist Zibotentan Induces Damage to the Nasal Olfactory Epithelium Possibly Mediated in Part through Type 2 Innate Lymphoid Cells. Toxicol. Pathol..

[33] Mathier M.A., Ishizawar D. (2010). Bosentan. Expert Opin. Pharmacother..

[34] Peacock A.J., Zamboni W., Vizza C.D. (2015). Ambrisentan for the treatment of adults with pulmonary arterial hypertension: A review. Curr. Med. Res. Opin..

[35] Chaumais M.C., Guignabert C., Savale L., Jais X., Boucly A., Montani D., Simonneau G., Humbert M., Sitbon O. (2015). Clinical pharmacology of endothelin receptor antagonists used in the treatment of pulmonary arterial hypertension. Am. J. Cardiovasc. Drugs.

[36] Dingemanse J., Sidharta P.N., Maddrey W.C., Rubin L.J., Mickail H. (2014). Efficacy, safety and clinical pharmacology of macitentan in comparison to other endothelin receptor antagonists in the treatment of pulmonary arterial hypertension. Expert Opin. Drug Saf..

[37] Bedan M., Grimm D., Wehland M., Simonsen U., Infanger M., Krüger M. (2018). A Focus on Macitentan in the Treatment of Pulmonary Arterial Hypertension. Basic Clin. Pharmacol. Toxicol..

[38] Frampton J.E. (2011). Ambrisentan. Am. J. Cardiovasc. Drugs.

[39] Croxtall J.D., Keam S.J. (2008). Ambrisentan. Drugs.

[40] Venitz J., Zack J., Gillies H., Allard M., Regnault J., Dufton C. (2012). Clinical pharmacokinetics and drug-drug interactions of endothelin receptor antagonists in pulmonary arterial hypertension. J. Clin. Pharmacol..

[41] European Medicines Agency Opsumit, INN-Macitentan, Annex. https://ec.europa.eu/health/documents/community-register/2013/20131220127396/anx_127396_en.pdf.

[42] Khadka A., Singh Brashier D.B., Tejus A., Sharma A.K. (2015). Macitentan: An important addition to the treatment of pulmonary arterial hypertension. J. Pharmacol. Pharmacother..

[43] Oldfield V., Lyseng-Williamson K.A. (2006). Bosentan: A review of its use in pulmonary arterial hypertension and systemic sclerosis. Am. J. Cardiovasc. Drugs.

[44] Benedict N., Seybert A., Mathier M.A. (2007). Evidence-based pharmacologic management of pulmonary arterial hypertension. Clin. Ther..

[45] Nagendran J., Sutendra G., Paterson I., Champion H.C., Webster L., Chiu B., Haromy A., Rebeyka I.M., Ross D.B., Michelakis E.D. (2013). Endothelin axis is upregulated in human and rat right ventricular hypertrophy. Circ. Res..

[46] Trow T.K., Taichman D.B. (2009). Endothelin receptor blockade in the management of pulmonary arterial hypertension: Selective and dual antagonism. Respir. Med..

[47] Dhillon S., Keating G.M. (2009). Bosentan: A review of its use in the management of mildly symptomatic pulmonary arterial hypertension. Am. J. Cardiovasc. Drugs.

[48] Galie N., Hoeper M.M., Simon J., Gibbs R., Simonneau G. (2011). Liver toxicity of sitaxentan in pulmonary arterial hypertension. Eur. Heart J..

[49] Montani D., Gunther S., Dorfmuller P., Perros F., Girerd B., Garcia G., Jais X., Savale L., Artaud-Macari E., Price L.C. (2013). Pulmonary arterial hypertension. Orphanet J. Rare Dis..

[50] Lai Y.C., Potoka K.C., Champion H.C., Mora A.L., Gladwin M.T. (2014). Pulmonary arterial hypertension: The clinical syndrome. Circ. Res..

[51] MacIver D.H., Adeniran I., MacIver I.R., Revell A., Zhang H. (2016). Physiological mechanisms of pulmonary hypertension. Am. Heart J..

[52] Prins K.W., Thenappan T. (2016). World Health Organization Group I Pulmonary Hypertension: Epidemiology and Pathophysiology. Cardiol. Clin..

[53] Klinger J.R., Elliott G., Levine D.J., Bossone E., Duvall L., Fagan K., Frantsve-Hawley J., Kawut S.M., Ryan J.J., Rosenzweig E.B. (2019). Therapy for Pulmonary Arterial Hypertension in Adults 2018: Update of the CHEST Guideline and Expert Panel Report. Chest.

[54] Rubin L.J., Badesch D.B., Barst R.J., Galie N., Black C.M., Keogh A., Pulido T., Frost A., Roux S., Leconte I. (2002). Bosentan therapy for pulmonary arterial hypertension. N. Engl. J. Med..

[55] Galie N., Olschewski H., Oudiz R.J., Torres F., Frost A., Ghofrani H.A., Badesch D.B., McGoon M.D., McLaughlin V.V., Roecker E.B. (2008). Ambrisentan for the treatment of pulmonary arterial hypertension: Results of the ambrisentan in pulmonary arterial hypertension, randomized, double-blind, placebo-controlled, multicenter, efficacy (ARIES) study 1 and 2. Circulation.

[56] Pulido T., Adzerikho I., Channick R.N., Delcroix M., Galie N., Ghofrani H.A., Jansa P., Jing Z.C., Le Brun F.O., Mehta S. (2013). Macitentan and morbidity and mortality in pulmonary arterial hypertension. N. Engl. J. Med..

[57] Duo-Ji M.M., Long Z.W. (2017). Comparative efficacy and acceptability of endothelin receptor antagonists for pulmonary arterial hypertension: A network meta-analysis. Int. J. Cardiol..

[58] D’Uscio L.V., Barton M., Shaw S., Moreau P., Luscher T.F. (1997). Structure and function of small arteries in salt-induced hypertension: Effects of chronic endothelin-subtype-A-receptor blockade. Hypertension.

[59] Barton M., d’Uscio L.V., Shaw S., Meyer P., Moreau P., Luscher T.F. (1998). ET(A) receptor blockade prevents increased tissue endothelin-1, vascular hypertrophy, and endothelial dysfunction in salt-sensitive hypertension. Hypertension.

[60] Bakris G.L., Lindholm L.H., Black H.R., Krum H., Linas S., Linseman J.V., Arterburn S., Sager P., Weber M. (2010). Divergent results using clinic and ambulatory blood pressures: Report of a darusentan-resistant hypertension trial. Hypertension.

[61] Weber M.A., Black H., Bakris G., Krum H., Linas S., Weiss R., Linseman J.V., Wiens B.L., Warren M.S., Lindholm L.H. (2009). A selective endothelin-receptor antagonist to reduce blood pressure in patients with treatment-resistant hypertension: A randomised, double-blind, placebo-controlled trial. Lancet.

[62] Black H.R., Bakris G.L., Weber M.A., Weiss R., Shahawy M.E., Marple R., Tannoury G., Linas S., Wiens B.L., Linseman J.V. (2007). Efficacy and safety of darusentan in patients with resistant hypertension: Results from a randomized, double-blind, placebo-controlled dose-ranging study. J. Clin. Hypertens Greenwich.

[63] Barton M., Haudenschild C.C., d’Uscio L.V., Shaw S., Munter K., Luscher T.F. (1998). Endothelin ETA receptor blockade restores NO-mediated endothelial function and inhibits atherosclerosis in apolipoprotein E-deficient mice. Proc. Natl. Acad. Sci. USA.

[64] Saitoh S., Matsumoto K., Kamioka M., Ohkawara H., Kaneshiro T., Ishibashi T., Maruyama Y. (2009). Novel pathway of endothelin-1 and reactive oxygen species in coronary vasospasm with endothelial dysfunction. Coron. Artery Dis..

[65] Kusuhara M., Yamaguchi K., Nagasaki K., Hayashi C., Suzaki A., Hori S., Handa S., Nakamura Y., Abe K. (1990). Production of endothelin in human cancer cell lines. Cancer Res..

[66] Peduto Eberl L., Bovey R., Juillerat-Jeanneret L. (2003). Endothelin-receptor antagonists are proapoptotic and antiproliferative in human colon cancer cells. Br. J. Cancer.

[67] Moody T.W., Ramos-Alvarez I., Moreno P., Mantey S.A., Ridnour L., Wink D., Jensen R.T. (2017). Endothelin causes transactivation of the EGFR and HER2 in non-small cell lung cancer cells. Peptides.

[68] Wang R., Dashwood R.H. (2011). Endothelins and their receptors in cancer: Identification of therapeutic targets. Pharmacol. Res..

[69] Bagnato A., Catt K.J. (1998). Endothelins as autocrine regulators of tumor cell growth. Trends Endocrinol. Metab..

[70] Harris A.L. (2002). Hypoxia—A key regulatory factor in tumour growth. Nat. Rev. Cancer.

[71] Smollich M., Gotte M., Kersting C., Fischgrabe J., Kiesel L., Wulfing P. (2008). Selective ETAR antagonist atrasentan inhibits hypoxia-induced breast cancer cell invasion. Breast Cancer Res. Treat..

[72] Masoud G.N., Li W. (2015). HIF-1α pathway: Role, regulation and intervention for cancer therapy. Acta Pharm. Sin. B.

[73] Knowles J., Loizidou M., Taylor I. (2005). Endothelin-1 and angiogenesis in cancer. Curr. Vasc. Pharmacol..

[74] Irani S., Salajegheh A., Smith R.A., Lam A.K. (2014). A review of the profile of endothelin axis in cancer and its management. Crit. Rev. Oncol. Hematol..

[75] Knowles J.P., Shi-Wen X., Haque S.U., Bhalla A., Dashwood M.R., Yang S., Taylor I., Winslet M.C., Abraham D.J., Loizidou M. (2012). Endothelin-1 stimulates colon cancer adjacent fibroblasts. Int. J. Cancer.

[76] Nelson J., Bagnato A., Battistini B., Nisen P. (2003). The endothelin axis: Emerging role in cancer. Nat. Rev. Cancer.

[77] Rosano L., Cianfrocca R., Spinella F., Di Castro V., Nicotra M.R., Lucidi A., Ferrandina G., Natali P.G., Bagnato A. (2011). Acquisition of chemoresistance and EMT phenotype is linked with activation of the endothelin A receptor pathway in ovarian carcinoma cells. Clin. Cancer Res..

[78] Coffman L., Mooney C., Lim J., Bai S., Silva I., Gong Y., Yang K., Buckanovich R.J. (2013). Endothelin receptor-A is required for the recruitment of antitumor T cells and modulates chemotherapy induction of cancer stem cells. Cancer Biol. Ther..

[79] Haque S.U., Dashwood M.R., Heetun M., Shiwen X., Farooqui N., Ramesh B., Welch H., Savage F.J., Ogunbiyi O., Abraham D.J. (2013). Efficacy of the specific endothelin a receptor antagonist zibotentan (ZD4054) in colorectal cancer: A preclinical study. Mol. Cancer Ther..

[80] Said N., Smith S., Sanchez-Carbayo M., Theodorescu D. (2011). Tumor endothelin-1 enhances metastatic colonization of the lung in mouse xenograft models of bladder cancer. J. Clin. Investig..

[81] Eltze E., Wild P.J., Wulfing C., Zwarthoff E.C., Burger M., Stoehr R., Korsching E., Hartmann A. (2009). Expression of the endothelin axis in noninvasive and superficially invasive bladder cancer: Relation to clinicopathologic and molecular prognostic parameters. Eur. Urol..

[82] Wulfing P., Diallo R., Kersting C., Wulfing C., Poremba C., Rody A., Greb R.R., Bocker W., Kiesel L. (2003). Expression of endothelin-1, endothelin-A, and endothelin-B receptor in human breast cancer and correlation with long-term follow-up. Clin. Cancer Res..

[83] Wulfing P., Kersting C., Tio J., Fischer R.J., Wulfing C., Poremba C., Diallo R., Bocker W., Kiesel L. (2004). Endothelin-1-, endothelin-A-, and endothelin-B-receptor expression is correlated with vascular endothelial growth factor expression and angiogenesis in breast cancer. Clin. Cancer Res..

[84] Sun D.J., Liu Y., Lu D.C., Kim W., Lee J.H., Maynard J., Deisseroth A. (2007). Endothelin-3 growth factor levels decreased in cervical cancer compared with normal cervical epithelial cells. Hum. Pathol..

[85] Hoosein M.M., Dashwood M.R., Dawas K., Ali H.M., Grant K., Savage F., Taylor I., Loizidou M. (2007). Altered endothelin receptor subtypes in colorectal cancer. Eur. J. Gastroenterol. Hepatol..

[86] Liakou P., Tepetes K., Germenis A., Leventaki V., Atsaves V., Patsouris E., Roidis N., Hatzitheophilou K., Rassidakis G.Z. (2012). Expression patterns of endothelin-1 and its receptors in colorectal cancer. J. Surg. Oncol..

[87] Fukui R., Nishimori H., Hata F., Yasoshima T., Ohno K., Yanai Y., Kamiguchi K., Denno R., Sato N., Hirata K. (2007). Inhibitory effect of endothelin A receptor blockade on tumor growth and liver metastasis of a human gastric cancer cell line. Gastric Cancer.

[88] Tao K., Wu C., Wu K., Li W., Han G., Shuai X., Wang G. (2012). Quantitative analysis of promoter methylation of the EDNRB gene in gastric cancer. Med. Oncol..

[89] Egidy G., Eberl L.P., Valdenaire O., Irmler M., Majdi R., Diserens A.C., Fontana A., Janzer R.C., Pinet F., Juillerat-Jeanneret L. (2000). The endothelin system in human glioblastoma. Lab. Investig..

[90] Vasaikar S., Tsipras G., Landazuri N., Costa H., Wilhelmi V., Scicluna P., Cui H.L., Mohammad A.A., Davoudi B., Shang M. (2018). Overexpression of endothelin B receptor in glioblastoma: A prognostic marker and therapeutic target?. BMC Cancer.

[91] Anguelova E., Beuvon F., Leonard N., Chaverot N., Varlet P., Couraud P.O., Daumas-Duport C., Cazaubon S. (2005). Functional endothelin ET B receptors are selectively expressed in human oligodendrogliomas. Brain Res. Mol. Brain Res..

[92] Ishimoto S., Wada K., Tanaka N., Yamanishi T., Ishihama K., Aikawa T., Okura M., Nakajima A., Kogo M., Kamisaki Y. (2012). Role of endothelin receptor signalling in squamous cell carcinoma. Int. J. Oncol..

[93] Wen Y.F., Qi B., Liu H., Mo H.Y., Chen Q.Y., Li J., Huang P.Y., Ye Y.F., Zhang Y., Deng M.Q. (2011). Polymorphisms in the endothelin-1 and endothelin a receptor genes and survival in patients with locoregionally advanced nasopharyngeal carcinoma. Clin. Cancer Res..

[94] Cong N., Li Z., Shao W., Li J., Yu S. (2016). Activation of ETA Receptor by Endothelin-1 Induces Hepatocellular Carcinoma Cell Migration and Invasion via ERK1/2 and AKT Signaling Pathways. J. Membr. Biol..

[95] Hsu L.S., Lee H.C., Chau G.Y., Yin P.H., Chi C.W., Lui W.Y. (2006). Aberrant methylation of EDNRB and p16 genes in hepatocellular carcinoma (HCC) in Taiwan. Oncol. Rep..

[96] Boldrini L., Gisfredi S., Ursino S., Faviana P., Lucchi M., Melfi F., Mussi A., Basolo F., Fontanini G. (2005). Expression of endothelin-1 is related to poor prognosis in non-small cell lung carcinoma. Eur. J. Cancer.

[97] Blouquit-Laye S., Regnier A., Beauchet A., Zimmermann U., Devillier P., Chinet T. (2010). Expression of endothelin receptor subtypes in bronchial tumors. Oncol. Rep..

[98] Demunter A., De Wolf-Peeters C., Degreef H., Stas M., van den Oord J.J. (2001). Expression of the endothelin-B receptor in pigment cell lesions of the skin. Evidence for its role as tumor progression marker in malignant melanoma. Virchows Arch..

[99] Smith S.L., Damato B.E., Scholes A.G., Nunn J., Field J.K., Heighway J. (2002). Decreased endothelin receptor B expression in large primary uveal melanomas is associated with early clinical metastasis and short survival. Br. J. Cancer.

[100] Bagnato A., Salani D., Di Castro V., Wu-Wong J.R., Tecce R., Nicotra M.R., Venuti A., Natali P.G. (1999). Expression of endothelin 1 and endothelin A receptor in ovarian carcinoma: Evidence for an autocrine role in tumor growth. Cancer Res..

[101] Rosano L., Spinella F., Di Castro V., Nicotra M.R., Dedhar S., de Herreros A.G., Natali P.G., Bagnato A. (2005). Endothelin-1 promotes epithelial-to-mesenchymal transition in human ovarian cancer cells. Cancer Res..

[102] Cook N., Brais R., Qian W., Hak C.C., Corrie P.G. (2015). Endothelin-1 and endothelin B receptor expression in pancreatic adenocarcinoma. J. Clin. Pathol..

[103] Gupta S., Prajapati A., Gulati M., Gautam S.K., Kumar S., Dalal V., Talmon G.A., Rachagani S., Jain M. (2020). Irreversible and sustained upregulation of endothelin axis during oncogene-associated pancreatic inflammation and cancer. Neoplasia.

[104] Bhargava S., Stummeyer T., Hotz B., Hines O.J., Reber H.A., Buhr H.J., Hotz H.G. (2005). Selective inhibition of endothelin receptor A as an anti-angiogenic and anti-proliferative strategy for human pancreatic cancer. J. Gastrointest. Surg..

[105] Godara G., Pecher S., Jukic D.M., D’Antonio J.M., Akhavan A., Nelson J.B., Pflug B.R. (2007). Distinct patterns of endothelin axis expression in primary prostate cancer. Urology.

[106] Nelson J.B., Hedican S.P., George D.J., Reddi A.H., Piantadosi S., Eisenberger M.A., Simons J.W. (1995). Identification of endothelin-1 in the pathophysiology of metastatic adenocarcinoma of the prostate. Nat. Med..

[107] Herrmann E., Eltze E., Bierer S., Bogemann M., Brinkmann O.A., Balnowair H., Hertle L., Wulfing C. (2007). Expression of the Endothelin-axis in the different histologic subtypes of renal cell carcinoma: A tissue microarray analysis. Oncol. Rep..

[108] Douglas M.L., Richardson M.M., Nicol D.L. (2004). Endothelin axis expression is markedly different in the two main subtypes of renal cell carcinoma. Cancer.

[109] Wuttig D., Zastrow S., Fussel S., Toma M.I., Meinhardt M., Kalman K., Junker K., Sanjmyatav J., Boll K., Hackermuller J. (2012). CD31, EDNRB and TSPAN7 are promising prognostic markers in clear-cell renal cell carcinoma revealed by genome-wide expression analyses of primary tumors and metastases. Int. J. Cancer.

[110] Yao M., Huang Y., Shioi K., Hattori K., Murakami T., Sano F., Baba M., Kondo K., Nakaigawa N., Kishida T. (2008). A three-gene expression signature model to predict clinical outcome of clear cell renal carcinoma. Int. J. Cancer.

[111] Irani S., Salajegheh A., Gopalan V., Smith R.A., Lam A.K. (2014). Expression profile of endothelin 1 and its receptor endothelin receptor A in papillary thyroid carcinoma and their correlations with clinicopathologic characteristics. Ann. Diagn. Pathol..

[112] Eltze E., Bertolin M., Korsching E., Wulfing P., Maggino T., Lelle R. (2007). Expression and prognostic relevance of endothelin-B receptor in vulvar cancer. Oncol. Rep..

[113] Askoxylakis V., Ferraro G.B., Badeaux M., Kodack D.P., Kirst I., Shankaraiah R.C., Wong C.S.F., Duda D.G., Fukumura D., Jain R.K. (2019). Dual endothelin receptor inhibition enhances T-DM1 efficacy in brain metastases from HER2-positive breast cancer. NPJ Breast Cancer.

[114] Kim S.J., Kim J.S., Kim S.W., Brantley E., Yun S.J., He J., Maya M., Zhang F., Wu Q., Lehembre F. (2011). Macitentan (ACT-064992), a tissue-targeting endothelin receptor antagonist, enhances therapeutic efficacy of paclitaxel by modulating survival pathways in orthotopic models of metastatic human ovarian cancer. Neoplasia.

[115] Lee H.J., Hanibuchi M., Kim S.J., Yu H., Kim M.S., He J., Langley R.R., Lehembre F., Regenass U., Fidler I.J. (2016). Treatment of experimental human breast cancer and lung cancer brain metastases in mice by macitentan, a dual antagonist of endothelin receptors, combined with paclitaxel. Neuro Oncol..

[116] Cianfrocca R., Rosano L., Tocci P., Sestito R., Caprara V., Di Castro V., De Maria R., Bagnato A. (2017). Blocking endothelin-1-receptor/beta-catenin circuit sensitizes to chemotherapy in colorectal cancer. Cell Death Differ..

[117] Goda K., Bacso Z., Szabo G. (2009). Multidrug resistance through the spectacle of P-glycoprotein. Curr. Cancer Drug Targets.

[118] Bradley G., Ling V. (1994). P-glycoprotein, multidrug resistance and tumor progression. Cancer Metastasis Rev..

[119] Hartz A.M., Bauer B., Fricker G., Miller D.S. (2004). Rapid regulation of P-glycoprotein at the blood-brain barrier by endothelin-1. Mol. Pharmacol..

[120] Yang K.M., Russell J., Lupu M.E., Cho H., Li X.-F., Koutcher J.A., Ling C.C. (2009). Atrasentan (ABT-627) enhances perfusion and reduces hypoxia in a human tumor xenograft model. Cancer Biol. Ther..

[121] Lalich M., McNeel D.G., Wilding G., Liu G. (2007). Endothelin receptor antagonists in cancer therapy. Cancer Investig..

[122] Nelson J.B., Fizazi K., Miller K., Higano C., Moul J.W., Akaza H., Morris T., McIntosh S., Pemberton K., Gleave M. (2012). Phase 3, randomized, placebo-controlled study of zibotentan (ZD4054) in patients with castration-resistant prostate cancer metastatic to bone. Cancer.

[123] Qi P., Chen M., Zhang L.X., Song R.X., He Z.H., Wang Z.P. (2015). A Meta-Analysis and Indirect Comparison of Endothelin A Receptor Antagonist for Castration-Resistant Prostate Cancer. PLoS ONE.

[124] Rosano L., Bagnato A. (2016). Endothelin therapeutics in cancer: Where are we?. Am. J. Physiol. Regul. Integr. Comp. Physiol..

[125] Miller K., Moul J.W., Gleave M., Fizazi K., Nelson J.B., Morris T., Nathan F.E., McIntosh S., Pemberton K., Higano C.S. (2013). Phase III, randomized, placebo-controlled study of once-daily oral zibotentan (ZD4054) in patients with non-metastatic castration-resistant prostate cancer. Prostate Cancer Prostatic Dis..

[126] Fizazi K., Higano C.S., Nelson J.B., Gleave M., Miller K., Morris T., Nathan F.E., McIntosh S., Pemberton K., Moul J.W. (2013). Phase III, randomized, placebo-controlled study of docetaxel in combination with zibotentan in patients with metastatic castration-resistant prostate cancer. J. Clin. Oncol..

[127] Chouaid C., Nathan F., Pemberton K., Morris T. (2011). A phase II, randomized, multicenter study to assess the efficacy, safety, and tolerability of zibotentan (ZD4054) in combination with pemetrexed in patients with advanced non-small cell lung cancer. Cancer Chemother. Pharmacol..

[128] Nelson J.B., Love W., Chin J.L., Saad F., Schulman C.C., Sleep D.J., Qian J., Steinberg J., Carducci M. (2008). Phase 3, randomized, controlled trial of atrasentan in patients with nonmetastatic, hormone-refractory prostate cancer. Cancer.

[129] Carducci M.A., Saad F., Abrahamsson P.A., Dearnaley D.P., Schulman C.C., North S.A., Sleep D.J., Isaacson J.D., Nelson J.B. (2007). A phase 3 randomized controlled trial of the efficacy and safety of atrasentan in men with metastatic hormone-refractory prostate cancer. Cancer.

[130] Quinn D.I., Tangen C.M., Hussain M., Lara P.N., Goldkorn A., Moinpour C.M., Garzotto M.G., Mack P.C., Carducci M.A., Monk J.P. (2013). Docetaxel and atrasentan versus docetaxel and placebo for men with advanced castration-resistant prostate cancer (SWOG S0421): A randomised phase 3 trial. Lancet Oncol..

[131] Lara P.N., Ely B., Quinn D.I., Mack P.C., Tangen C., Gertz E., Twardowski P.W., Goldkorn A., Hussain M., Vogelzang N.J. (2014). Serum biomarkers of bone metabolism in castration-resistant prostate cancer patients with skeletal metastases: Results from SWOG 0421. J. Natl. Cancer Inst..

[132] Goldkorn A., Ely B., Quinn D.I., Tangen C.M., Fink L.M., Xu T., Twardowski P., Van Veldhuizen P.J., Agarwal N., Carducci M.A. (2014). Circulating tumor cell counts are prognostic of overall survival in SWOG S0421: A phase III trial of docetaxel with or without atrasentan for metastatic castration-resistant prostate cancer. J. Clin. Oncol..

[133] Carducci M.A., Manola J., Nair S.G., Liu G., Rousey S., Dutcher J.P., Wilding G. (2015). Atrasentan in Patients With Advanced Renal Cell Carcinoma: A Phase 2 Trial of the ECOG-ACRIN Cancer Research Group (E6800). Clin. Genitourin. Cancer.

[134] Kim R., Chiorean E.G., Amin M., Rocha-Lima C.M.S., Gandhi J., Harris W.P., Song T., Portnoy D. (2017). Phase 2 study of combination SPI-1620 with docetaxel as second-line advanced biliary tract cancer treatment. Br. J. Cancer.

[135] Kefford R., Beith J.M., Van Hazel G.A., Millward M., Trotter J.M., Wyld D.K., Kusic R., Shreeniwas R., Morganti A., Ballmer A. (2007). A phase II study of bosentan, a dual endothelin receptor antagonist, as monotherapy in patients with stage IV metastatic melanoma. Investig. New Drugs.

[136] Kefford R.F., Clingan P.R., Brady B., Ballmer A., Morganti A., Hersey P. (2010). A randomized, double-blind, placebo-controlled study of high-dose bosentan in patients with stage IV metastatic melanoma receiving first-line dacarbazine chemotherapy. Mol. Cancer.

[137] De Jong M., Maina T. (2010). Of mice and humans: Are they the same? Implications in cancer translational research. J. Nucl. Med..

[138] Pollock J.S., Pollock D.M. (2019). SONAR propels endothelin A receptor antagonists to success. Nat. Rev. Nephrol..

[139] Kohan D.E., Barton M. (2014). Endothelin and endothelin antagonists in chronic kidney disease. Kidney Int..

[140] Mann J.F., Green D., Jamerson K., Ruilope L.M., Kuranoff S.J., Littke T., Viberti G. (2010). Avosentan for overt diabetic nephropathy. J. Am. Soc. Nephrol..

[141] Kohan D.E., Pritchett Y., Molitch M., Wen S., Garimella T., Audhya P., Andress D.L. (2011). Addition of atrasentan to renin-angiotensin system blockade reduces albuminuria in diabetic nephropathy. J. Am. Soc. Nephrol..

[142] Heerspink H.J.L., Parving H.H., Andress D.L., Bakris G., Correa-Rotter R., Hou F.F., Kitzman D.W., Kohan D., Makino H., McMurray J.J.V. (2019). Atrasentan and renal events in patients with type 2 diabetes and chronic kidney disease (SONAR): A double-blind, randomised, placebo-controlled trial. Lancet.

[143] Dhaun N., MacIntyre I.M., Kerr D., Melville V., Johnston N.R., Haughie S., Goddard J., Webb D.J. (2011). Selective endothelin-A receptor antagonism reduces proteinuria, blood pressure, and arterial stiffness in chronic proteinuric kidney disease. Hypertension.

[144] Trachtman H., Nelson P., Adler S., Campbell K.N., Chaudhuri A., Derebail V.K., Gambaro G., Gesualdo L., Gipson D.S., Hogan J. (2018). DUET: A Phase 2 Study Evaluating the Efficacy and Safety of Sparsentan in Patients with FSGS. J. Am. Soc. Nephrol..

[145] Dhaun N., Melville V., Blackwell S., Talwar D.K., Johnston N.R., Goddard J., Webb D.J. (2013). Endothelin-A receptor antagonism modifies cardiovascular risk factors in CKD. J. Am. Soc. Nephrol..

[146] Lin C.-W., Mostafa N.M., Andress D.L., Brennan J.J., Klein C.E., Awni W.M. (2018). Relationship Between Atrasentan Concentrations and Urinary Albumin to Creatinine Ratio in Western and Japanese Patients With Diabetic Nephropathy. Clin. Ther..

[147] De Zeeuw D., Coll B., Andress D., Brennan J.J., Tang H., Houser M., Correa-Rotter R., Kohan D., Lambers Heerspink H.J., Makino H. (2014). The endothelin antagonist atrasentan lowers residual albuminuria in patients with type 2 diabetic nephropathy. J. Am. Soc. Nephrol..

[148] Kohan D.E., Lambers Heerspink H.J., Coll B., Andress D., Brennan J.J., Kitzman D.W., Correa-Rotter R., Makino H., Perkovic V., Hou F.F. (2015). Predictors of Atrasentan-Associated Fluid Retention and Change in Albuminuria in Patients with Diabetic Nephropathy. Clin. J. Am. Soc. Nephrol..

[149] Perez-Gomez M.V., Sanchez-Nino M.D., Sanz A.B., Martin-Cleary C., Ruiz-Ortega M., Egido J., Navarro-Gonzalez J.F., Ortiz A., Fernandez-Fernandez B. (2015). Horizon 2020 in Diabetic Kidney Disease: The Clinical Trial Pipeline for Add-On Therapies on Top of Renin Angiotensin System Blockade. J. Clin. Med..

[150] King T.E., Brown K.K., Raghu G., du Bois R.M., Lynch D.A., Martinez F., Valeyre D., Leconte I., Morganti A., Roux S. (2011). BUILD-3: A randomized, controlled trial of bosentan in idiopathic pulmonary fibrosis. Am. J. Respir. Crit. Care Med..

[151] King T.E., Behr J., Brown K.K., du Bois R.M., Lancaster L., de Andrade J.A., Stahler G., Leconte I., Roux S., Raghu G. (2008). BUILD-1: A randomized placebo-controlled trial of bosentan in idiopathic pulmonary fibrosis. Am. J. Respir. Crit. Care Med..

[152] Raghu G., Behr J., Brown K.K., Egan J.J., Kawut S.M., Flaherty K.R., Martinez F.J., Nathan S.D., Wells A.U., Collard H.R. (2013). Treatment of idiopathic pulmonary fibrosis with ambrisentan: A parallel, randomized trial. Ann. Intern. Med..

[153] Raghu G., Million-Rousseau R., Morganti A., Perchenet L., Behr J. (2013). Macitentan for the treatment of idiopathic pulmonary fibrosis: The randomised controlled MUSIC trial. Eur. Respir. J..

[154] Macdonald R.L., Kassell N.F., Mayer S., Ruefenacht D., Schmiedek P., Weidauer S., Frey A., Roux S., Pasqualin A. (2008). Clazosentan to overcome neurological ischemia and infarction occurring after subarachnoid hemorrhage (CONSCIOUS-1): Randomized, double-blind, placebo-controlled phase 2 dose-finding trial. Stroke.

[155] Macdonald R.L., Higashida R.T., Keller E., Mayer S.A., Molyneux A., Raabe A., Vajkoczy P., Wanke I., Bach D., Frey A. (2011). Clazosentan, an endothelin receptor antagonist, in patients with aneurysmal subarachnoid haemorrhage undergoing surgical clipping: A randomised, double-blind, placebo-controlled phase 3 trial (CONSCIOUS-2). Lancet Neurol..

[156] Macdonald R.L., Higashida R.T., Keller E., Mayer S.A., Molyneux A., Raabe A., Vajkoczy P., Wanke I., Bach D., Frey A. (2012). Randomized trial of clazosentan in patients with aneurysmal subarachnoid hemorrhage undergoing endovascular coiling. Stroke.

[157] Khanna D., Denton C.P., Merkel P.A., Krieg T., Le Brun F.O., Marr A., Papadakis K., Pope J., Matucci-Cerinic M., Furst D.E. (2016). Effect of Macitentan on the Development of New Ischemic Digital Ulcers in Patients With Systemic Sclerosis: DUAL-1 and DUAL-2 Randomized Clinical Trials. JAMA.

[158] Korn J.H., Mayes M., Matucci Cerinic M., Rainisio M., Pope J., Hachulla E., Rich E., Carpentier P., Molitor J., Seibold J.R. (2004). Digital ulcers in systemic sclerosis: Prevention by treatment with bosentan, an oral endothelin receptor antagonist. Arthritis Rheum..

[159] Matucci-Cerinic M., Denton C.P., Furst D.E., Mayes M.D., Hsu V.M., Carpentier P., Wigley F.M., Black C.M., Fessler B.J., Merkel P.A. (2011). Bosentan treatment of digital ulcers related to systemic sclerosis: Results from the RAPIDS-2 randomised, double-blind, placebo-controlled trial. Ann. Rheum. Dis..

[160] Rezus E., Burlui A.M., Gafton B., Stratulat T.A., Zota G.R., Cardoneanu A., Rezus C. (2020). A patient-centered approach to the burden of symptoms in patients with scleroderma treated with Bosentan: A prospective single-center observational study. Exp. Ther. Med..

[161] Farrah T.E., Anand A., Gallacher P.J., Kimmitt R., Carter E., Dear J.W., Mills N.L., Webb D.J., Dhaun N. (2019). Endothelin Receptor Antagonism Improves Lipid Profiles and Lowers PCSK9 (Proprotein Convertase Subtilisin/Kexin Type 9) in Patients With Chronic Kidney Disease. Hypertension.

[162] Phelan M., Perrine S.P., Brauer M., Faller D.V. (1995). Sickle erythrocytes, after sickling, regulate the expression of the endothelin-1 gene and protein in human endothelial cells in culture. J. Clin. Investig..

[163] Tharaux P.-L., Hagège I., Placier S., Vayssairat M., Kanfer A., Girot R., Dussaule J.-C. (2005). Urinary endothelin-1 as a marker of renal damage in sickle cell disease. Nephrol. Dial. Transplant..

[164] Sabaa N., de Franceschi L., Bonnin P., Castier Y., Malpeli G., Debbabi H., Galaup A., Maier-Redelsperger M., Vandermeersch S., Scarpa A. (2008). Endothelin receptor antagonism prevents hypoxia-induced mortality and morbidity in a mouse model of sickle-cell disease. J. Clin. Investig..

[165] Kasztan M., Fox B.M., Speed J.S., De Miguel C., Gohar E.Y., Townes T.M., Kutlar A., Pollock J.S., Pollock D.M. (2017). Long-Term Endothelin-A Receptor Antagonism Provides Robust Renal Protection in Humanized Sickle Cell Disease Mice. J. Am. Soc. Nephrol..

[166] Wynn T.A. (2008). Cellular and molecular mechanisms of fibrosis. J. Pathol..

[167] Katwa L.C., Guarda E., Weber K.T. (1993). Endothelin receptors in cultured adult rat cardiac fibroblasts. Cardiovasc. Res..

[168] Clozel M., Salloukh H. (2005). Role of endothelin in fibrosis and anti-fibrotic potential of bosentan. Ann. Med..

[169] Poncet S., Meyer S., Richard C., Aubert J.D., Juillerat-Jeanneret L. (2005). The expression and function of the endothelin system in contractile properties of vaginal myofibroblasts of women with uterovaginal prolapse. Am. J. Obstet. Gynecol..

[170] Xu S., Denton C.P., Holmes A., Dashwood M.R., Abraham D.J., Black C.M. (1998). Endothelins: Effect on matrix biosynthesis and proliferation in normal and scleroderma fibroblasts. J. Cardiovasc. Pharmacol..

[171] Rizvi M.A., Katwa L., Spadone D.P., Myers P.R. (1996). The effects of endothelin-1 on collagen type I and type III synthesis in cultured porcine coronary artery vascular smooth muscle cells. J. Mol. Cell Cardiol..

[172] Marini M., Carpi S., Bellini A., Patalano F., Mattoli S. (1996). Endothelin-1 induces increased fibronectin expression in human bronchial epithelial cells. Biochem. Biophys. Res. Commun..

[173] Shi-Wen X., Denton C.P., Dashwood M.R., Holmes A.M., Bou-Gharios G., Pearson J.D., Black C.M., Abraham D.J. (2001). Fibroblast matrix gene expression and connective tissue remodeling: Role of endothelin-1. J. Investig. Dermatol..

[174] Xu S.W., Howat S.L., Renzoni E.A., Holmes A., Pearson J.D., Dashwood M.R., Bou-Gharios G., Denton C.P., du Bois R.M., Black C.M. (2004). Endothelin-1 induces expression of matrix-associated genes in lung fibroblasts through MEK/ERK. J. Biol. Chem..

[175] Feng H.Q., Weymouth N.D., Rockey D.C. (2009). Endothelin antagonism in portal hypertensive mice: Implications for endothelin receptor-specific signaling in liver disease. Am. J. Physiol. Gastrointest. Liver Physiol..

[176] Rockey D.C., Chung J.J. (1996). Endothelin antagonism in experimental hepatic fibrosis. Implications for endothelin in the pathogenesis of wound healing. J. Clin. Investig..

[177] Antoniu S.A. (2008). Targeting the endothelin pathway in the idiopathic pulmonary fibrosis: The role of bosentan. Expert Opin. Ther. Targets.

[178] Weng C.M., Yu C.C., Kuo M.L., Chen B.C., Lin C.H. (2014). Endothelin-1 induces connective tissue growth factor expression in human lung fibroblasts by ETAR-dependent JNK/AP-1 pathway. Biochem. Pharmacol..

[179] Wind S., Schmid U., Freiwald M., Marzin K., Lotz R., Ebner T., Stopfer P., Dallinger C. (2019). Clinical Pharmacokinetics and Pharmacodynamics of Nintedanib. Clin. Pharmacokinet..

[180] Park S.H., Saleh D., Giaid A., Michel R.P. (1997). Increased endothelin-1 in bleomycin-induced pulmonary fibrosis and the effect of an endothelin receptor antagonist. Am. J. Respir. Crit. Care Med..

[181] King T.E. (2008). Bosentan for idiopathic pulmonary fibrosis. Curr. Opin. Investig. Drugs.

[182] Zimmermann M., Seifert V. (1998). Endothelin and subarachnoid hemorrhage: An overview. Neurosurgery.

[183] Chow M., Dumont A.S., Kassell N.F. (2002). Endothelin receptor antagonists and cerebral vasospasm: An update. Neurosurgery.

[184] Macdonald R.L. (2008). Clazosentan: An endothelin receptor antagonist for treatment of vasospasm after subarachnoid hemorrhage. Expert Opin. Investig. Drugs.

[185] Armstead W.M. (2004). Endothelins and the role of endothelin antagonists in the management of posttraumatic vasospasm. Curr. Pharm. Des..

[186] Mayer Stephan A., Aldrich E.F., Bruder N., Hmissi A., Macdonald R.L., Viarasilpa T., Marr A., Roux S., Higashida Randall T. (2019). Thick and Diffuse Subarachnoid Blood as a Treatment Effect Modifier of Clazosentan After Subarachnoid Hemorrhage. Stroke.

[187] Arefiev K., Fiorentino D.F., Chung L. (2011). Endothelin Receptor Antagonists for the Treatment of Raynaud’s Phenomenon and Digital Ulcers in Systemic Sclerosis. Int. J. Rheumatol..

[188] Cozzani E., Javor S., Laborai E., Drosera M., Parodi A. (2013). Endothelin-1 levels in scleroderma patients: A pilot study. ISRN Dermatol..

[189] Yamane K., Miyauchi T., Suzuki N., Yuhara T., Akama T., Suzuki H., Kashiwagi H. (1992). Significance of plasma endothelin-1 levels in patients with systemic sclerosis. J. Rheumatol..

[190] Zamora M.R., O’Brien R.F., Rutherford R.B., Weil J.V. (1990). Serum endothelin-1 concentrations and cold provocation in primary Raynaud’s phenomenon. Lancet.

[191] Kahaleh M.B. (1991). Endothelin, an endothelial-dependent vasoconstrictor in scleroderma. Enhanced production and profibrotic action. Arthritis Rheum..

[192] Richard V., Solans V., Favre J., Henry J.P., Lallemand F., Thuillez C., Marie I. (2008). Role of endogenous endothelin in endothelial dysfunction in murine model of systemic sclerosis: Tight skin mice 1. Fundam. Clin. Pharmacol..

[193] Avouac J., Riemekasten G., Meune C., Ruiz B., Kahan A., Allanore Y. (2015). Autoantibodies against Endothelin 1 Type A Receptor Are Strong Predictors of Digital Ulcers in Systemic Sclerosis. J. Rheumatol..

[194] Kowal-Bielecka O., Fransen J., Avouac J., Becker M., Kulak A., Allanore Y., Distler O., Clements P., Cutolo M., Czirjak L. (2017). Update of EULAR recommendations for the treatment of systemic sclerosis. Ann. Rheum. Dis..

[195] Kabunga P., Coghlan G. (2008). Endothelin receptor antagonism: Role in the treatment of pulmonary arterial hypertension related to scleroderma. Drugs.

[196] Shetty N., Derk C.T. (2011). Endothelin receptor antagonists as disease modifiers in systemic sclerosis. Inflamm. Allergy Drug Targets.

[197] Raffa R.B., Schupsky J.J., Martinez R.P., Jacoby H.I. (1991). Endothelin-1-induced nociception. Life Sci..

[198] Piovezan A.P., D’Orleans-Juste P., Tonussi C.R., Rae G.A. (1998). Effects of endothelin-1 on capsaicin-induced nociception in mice. Eur. J. Pharmacol..

[199] Khodorova A., Montmayeur J.P., Strichartz G. (2009). Endothelin receptors and pain. J. Pain.

[200] Smith T.P., Haymond T., Smith S.N., Sweitzer S.M. (2014). Evidence for the endothelin system as an emerging therapeutic target for the treatment of chronic pain. J. Pain Res..

[201] Furukawa A., Shinoda M., Kubo A., Honda K., Akasaka R., Yonehara Y., Iwata K. (2018). Endothelin Signaling Contributes to Modulation of Nociception in Early-stage Tongue Cancer in Rats. Anesthesiology.

[202] Tang Y., Peng H., Liao Q., Gan L., Zhang R., Huang L., Ding Z., Yang H., Yan X., Gu Y. (2016). Study of breakthrough cancer pain in an animal model induced by endothelin-1. Neurosci. Lett..

[203] Lutz B.M., Wu S., Gu X., Atianjoh F.E., Li Z., Fox B.M., Pollock D.M., Tao Y.X. (2018). Endothelin type A receptors mediate pain in a mouse model of sickle cell disease. Haematologica.

[204] Selenko-Gebauer N., Duschek N., Minimair G., Stingl G., Karlhofer F. (2006). Successful treatment of patients with severe secondary Raynaud’s phenomenon with the endothelin receptor antagonist bosentan. Rheumatology Oxford.

[205] Williams J.T., Christie M.J., Manzoni O. (2001). Cellular and synaptic adaptations mediating opioid dependence. Physiol. Rev..

[206] Matsumura K., Abe I., Fukuhara M., Tominaga M., Tsuchihashi T., Kobayashi K., Fujishima M. (1994). Naloxone augments sympathetic outflow induced by centrally administered endothelin in conscious rabbits. Am. J. Physiol..

[207] Modanlou H.D., Beharry K. (1998). Biochemical and molecular endothelin responses to morphine sulfate infusion in conscious newborn piglets. Can. J. Physiol. Pharmacol..

[208] Puppala B.L., Matwyshyn G., Bhalla S., Gulati A. (2004). Evidence that morphine tolerance may be regulated by endothelin in the neonatal rat. Biol. Neonate.

[209] Bhalla S., Matwyshyn G., Gulati A. (2005). Morphine tolerance does not develop in mice treated with endothelin-A receptor antagonists. Brain Res..

[210] Quang P.N., Schmidt B.L. (2010). Endothelin-A receptor antagonism attenuates carcinoma-induced pain through opioids in mice. J. Pain.

[211] Bhalla S., Pais G., Tapia M., Gulati A. (2015). Endothelin ETA receptor antagonist reverses naloxone-precipitated opioid withdrawal in mice. Can. J. Physiol. Pharmacol..

[212] Kohan D.E., Cleland J.G., Rubin L.J., Theodorescu D., Barton M. (2012). Clinical trials with endothelin receptor antagonists: What went wrong and where can we improve?. Life Sci..

[213] Gulati A., Sunila E.S., Kuttan G. (2012). IRL-1620, an endothelin-B receptor agonist, enhanced radiation induced reduction in tumor volume in Dalton’s Lymphoma Ascites tumor model. Arzneimittelforschung.

[214] Ji L., Dong C., Fan R., Qi S. (2020). A high affinity nanobody against endothelin receptor type B: A new approach to the treatment of melanoma. Mol. Biol. Rep..

[215] Mangat G.S., Jaggi A.S., Singh N. (2014). Ameliorative Effect of a Selective Endothelin ETA Receptor Antagonist in Rat Model of L-Methionine-induced Vascular Dementia. Korean J. Physiol. Pharmacol..

[216] Singh P., Gupta S., Sharma B. (2016). Antagonism of Endothelin (ETA and ETB) Receptors During Renovascular Hypertension-Induced Vascular Dementia Improves Cognition. Curr. Neurovasc. Res..

